# Supplementary figures and images for: Association between socioeconomic status and cardiovascular disease by sex: Mediating roles of psychological and behavioral factors
Source: PLoS One. 2026 Apr 1;21(4):e0345573. doi: 10.1371/journal.pone.0345573 (PMC13042698; doi:10.1371/journal.pone.0345573)

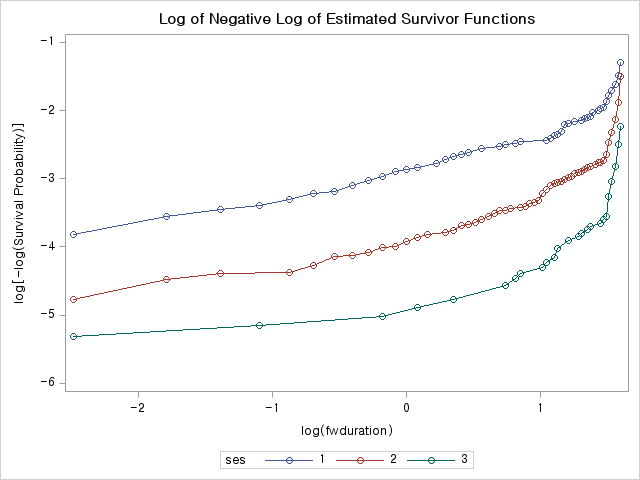

Supplement: S1 Fig — Log of the negative log of the estimated survivor function plotted against the log of follow-up duration for men, stratified by SES group (three categories). (TIFF) [file pone.0345573.s001.tiff]

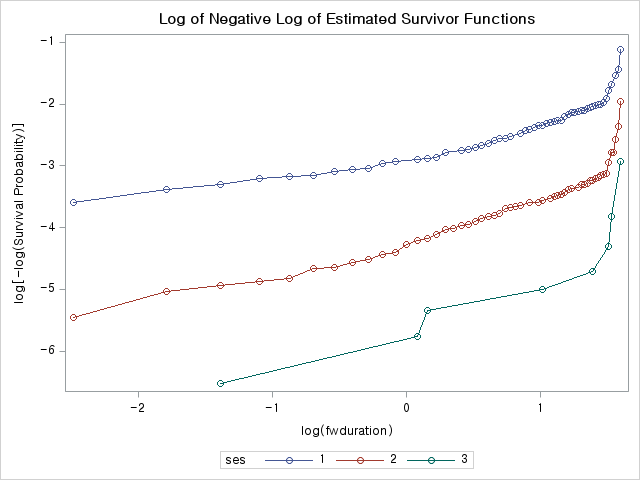

Supplement: S2 Fig — Log of the negative log of the estimated survivor function plotted against the log of follow-up duration for women, stratified by SES group (three categories). (TIFF) [file pone.0345573.s002.tiff]

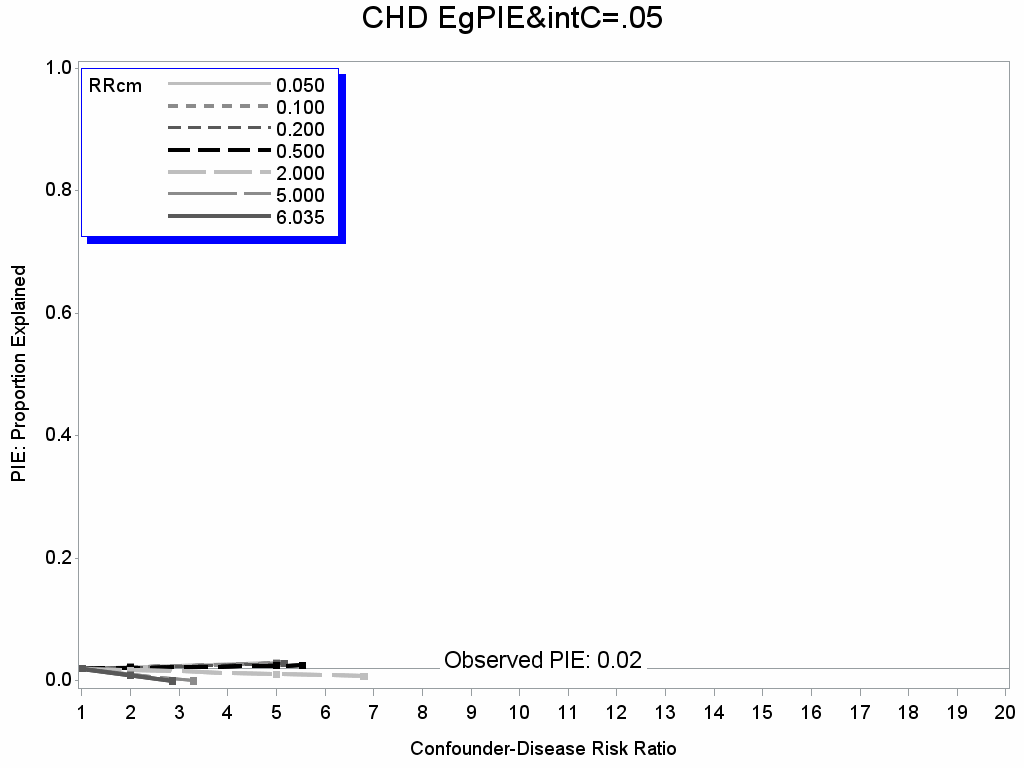

Supplement: S3 Fig — Each line depicts the adjusted PIE that aligns with the observed PIE under specific confounder–outcome and confounder–mediator (RRCM) risk ratios. (TIFF) [file pone.0345573.s003.tiff]

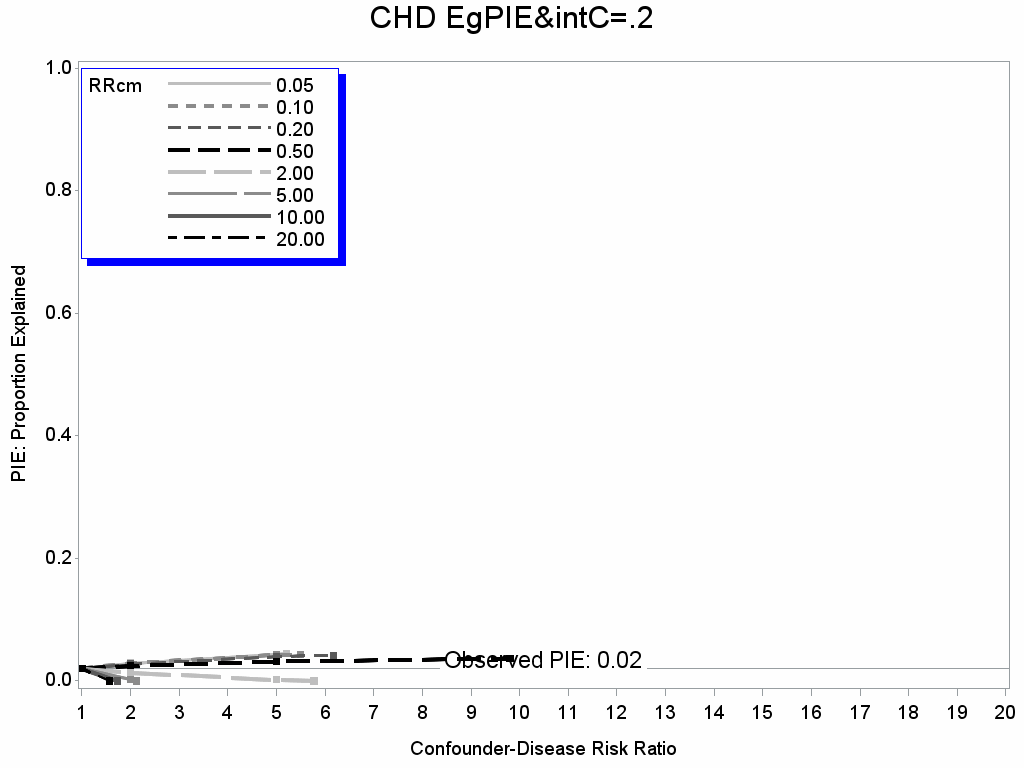

Supplement: S4 Fig — Each line depicts the adjusted PIE that aligns with the observed PIE under specific confounder–outcome and confounder–mediator (RRCM) risk ratios. (TIFF) [file pone.0345573.s004.tiff]

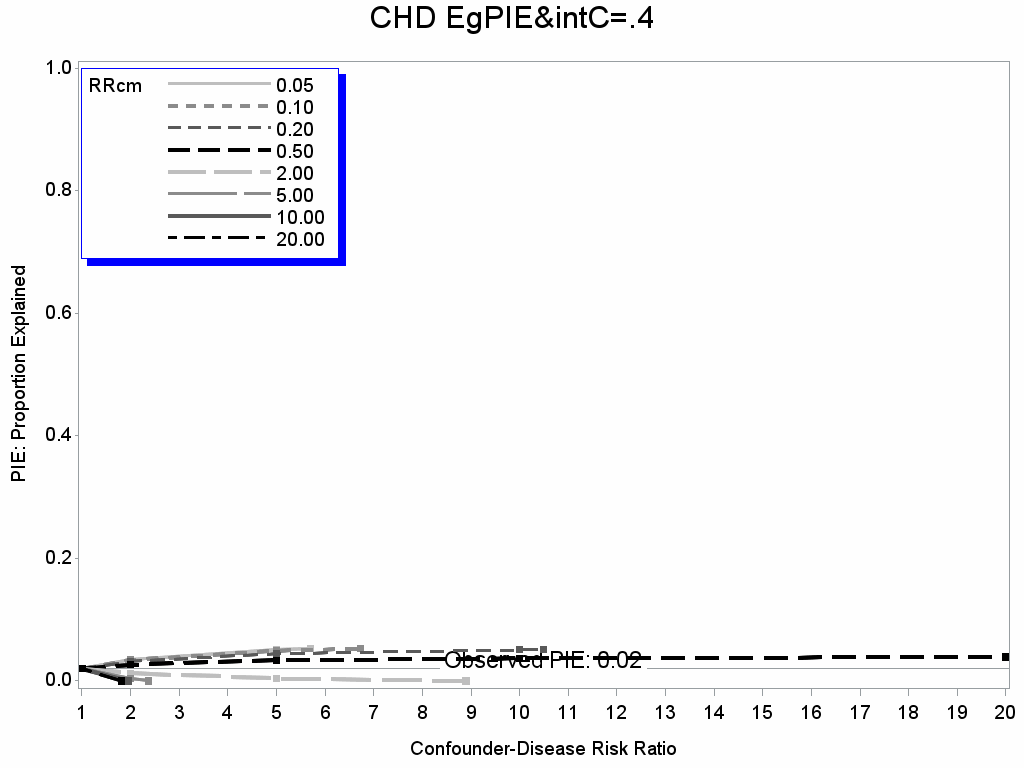

Supplement: S5 Fig — Each line depicts the adjusted PIE that aligns with the observed PIE under specific confounder–outcome and confounder–mediator (RRCM) risk ratios. (TIFF) [file pone.0345573.s005.tiff]

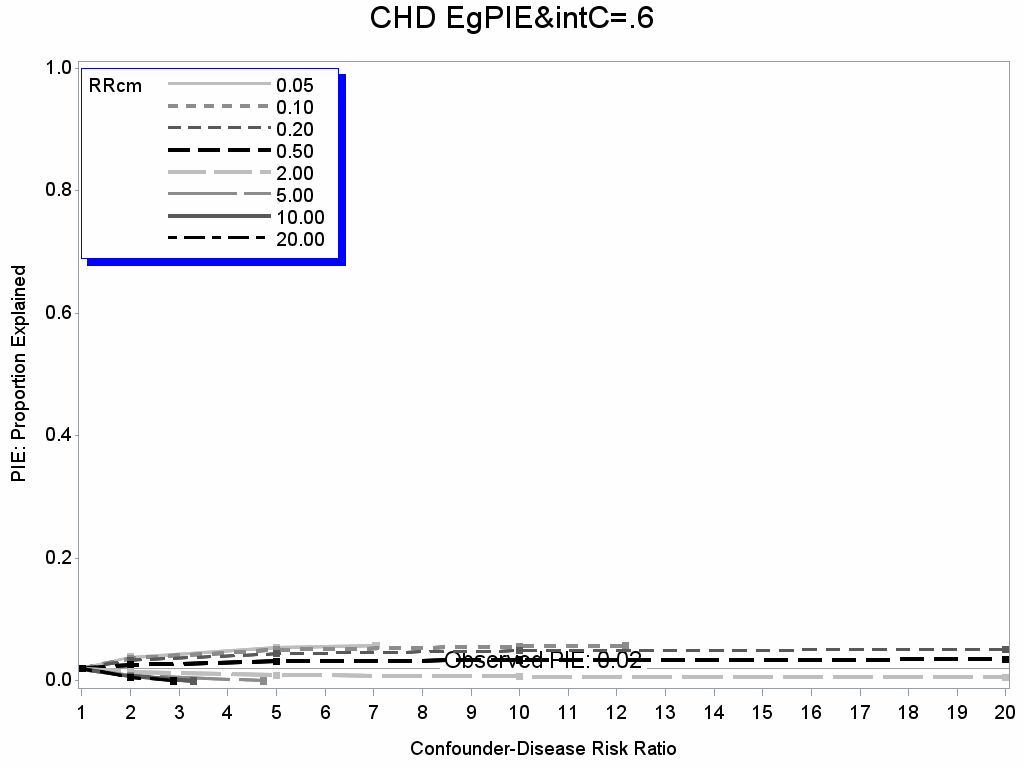

Supplement: S6 Fig — Each line depicts the adjusted PIE that aligns with the observed PIE under specific confounder–outcome and confounder–mediator (RRCM) risk ratios. (TIFF) [file pone.0345573.s006.tiff]

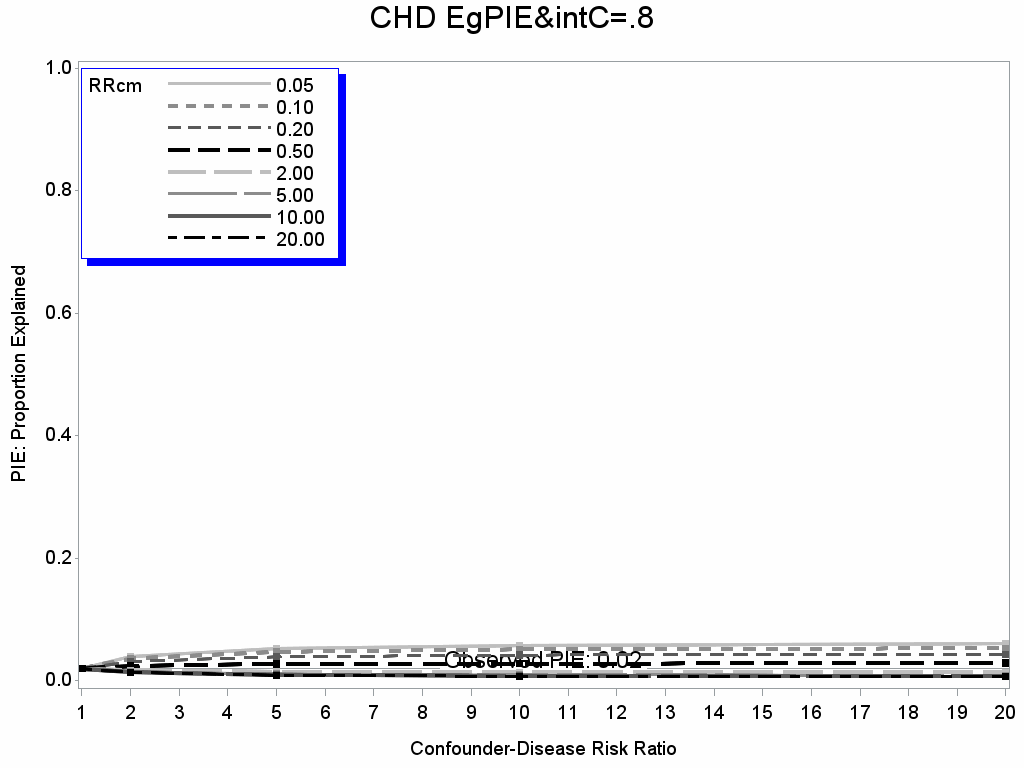

Supplement: S7 Fig — Each line depicts the adjusted PIE that aligns with the observed PIE under specific confounder–outcome and confounder–mediator (RRCM) risk ratios. (TIFF) [file pone.0345573.s007.tiff]

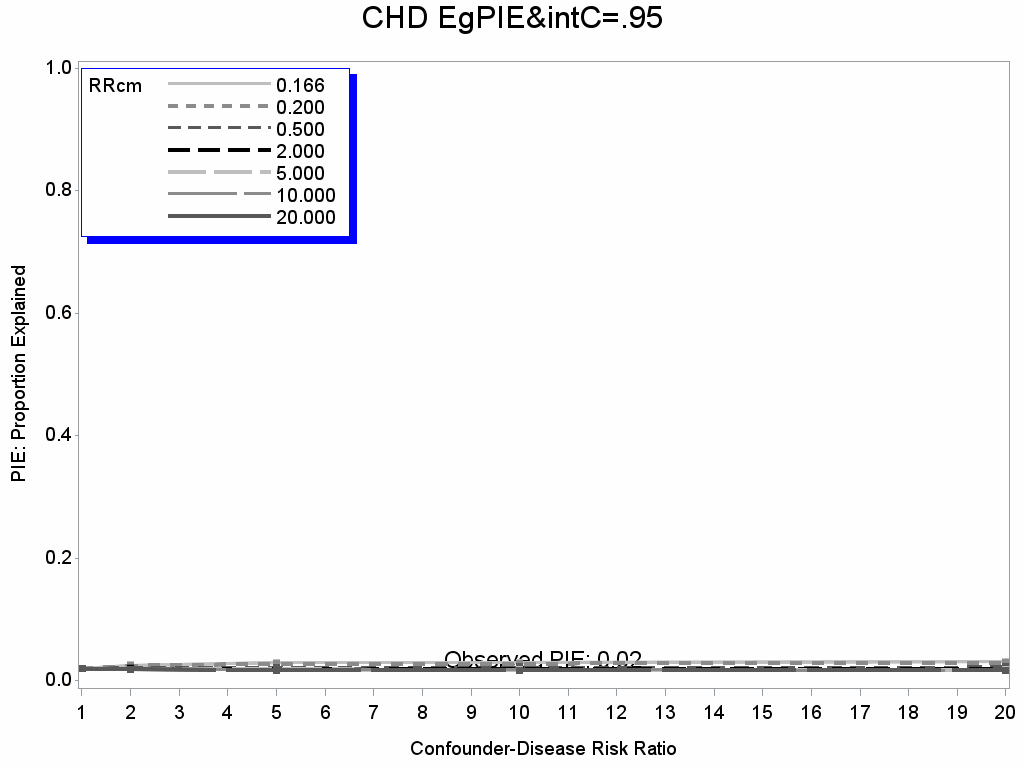

Supplement: S8 Fig — Each line depicts the adjusted PIE that aligns with the observed PIE under specific confounder–outcome and confounder–mediator (RRCM) risk ratios. (TIFF) [file pone.0345573.s008.tiff]
