# Supplementary material for: Association between socioeconomic status and cardiovascular disease by sex: Mediating roles of psychological and behavioral factors
Source: PLoS One. 2026 Apr 1;21(4):e0345573. doi: 10.1371/journal.pone.0345573 (PMC13042698; doi:10.1371/journal.pone.0345573)
Supplement: S1 Table — Abbreviation: DF, degree of freedom; AIC, Akaike information criterion; BIC, Bayesian information criterion; ABIC, Adjusted BIC. (DOCX) [file pone.0345573.s009.docx]

**S1 Table. Summary of model fit statistics for different numbers of latent classes by sex.**

| **Number of classes** | **DF** | **G_SQUARED** | **AIC** | **BIC** | **ABIC** | **Entropy** |
| --- | --- | --- | --- | --- | --- | --- |
| **Men** |  |  |  |  |  |  |
| 2 | 63 | 566.2 | 600.2 | 711.1 | 657.1 | 0.58 |
| **3** | **54** | **90.7** | **142.7** | **312.3** | **229.7** | **0.63** |
| 4 | 45 | 42.7 | 112.7 | 341.1 | 229.9 | 0.54 |
| 5 | 36 | 28.8 | 116.8 | 403.9 | 264.1 | 0.57 |
| **Women** |  |  |  |  |  |  |
| 2 | 63 | 681.9 | 715.9 | 830.5 | 776.4 | 0.57 |
| **3** | **54** | **150.3** | **202.3** | **377.4** | **294.8** | **0.69** |
| 4 | 45 | 69.3 | 139.3 | 375.1 | 263.9 | 0.66 |
| 5 | 36 | 14.8 | 102.8 | 399.2 | 259.4 | 0.58 |

Abbreviation: DF, degree of freedom; AIC, Akaike information criterion; BIC, Bayesian information criterion; ABIC, Adjusted BIC.
