# Supplementary material for: Association between socioeconomic status and cardiovascular disease by sex: Mediating roles of psychological and behavioral factors
Source: PLoS One. 2026 Apr 1;21(4):e0345573. doi: 10.1371/journal.pone.0345573 (PMC13042698; doi:10.1371/journal.pone.0345573)
Supplement: S2 Table — Abbreviations: AIC, Akaike information criterion. (DOCX) [file pone.0345573.s010.docx]

**S2 Table. Goodness of fit of the exponential and Weibull distribution.**

|  | **AIC** | |
| --- | --- | --- |
|  | Men | Women |
| Exponential | 3225.1 | 3515.9 |
| Weibull | 3145.1 | 3474.8 |

Abbreviations: AIC, Akaike information criterion.
