# Supplementary material for: Association between socioeconomic status and cardiovascular disease by sex: Mediating roles of psychological and behavioral factors
Source: PLoS One. 2026 Apr 1;21(4):e0345573. doi: 10.1371/journal.pone.0345573 (PMC13042698; doi:10.1371/journal.pone.0345573)
Supplement: S3 Table — * p < .05. ** p < .01. ***p < .001. Note: The model was adjusted for age, residence, marital status, obesity, previous diabetes mellitus, and previous hypertension. The measure of effect modification on the multiplicative scale is expressed as a time ratio (TR), derived by exponentiating the regression coefficients. The measure of effect modification on the additive scale was assessed using the relative excess risk due to interaction (RERI), calculated by converting TR into hazard ratios (HR) based on the parameter scale, and interpreting HR as an approximation of risk ratios (RR). Confidence intervals were estimated using the delta method. (DOCX) [file pone.0345573.s011.docx]

**S3 Table. Effect modification on multiplicative and additive scales between socioeconomic status and binary mediators by sex.**

| **Measure of effect modification on multiplicative scale** | | | | |
| --- | --- | --- | --- | --- |
|  | Men | | Women | |
|  | Time ratio  (95% CI) | P-value | Time ratio  (95% CI) | P-value |
| Low SES: having depressed mood | 1.22  (0.75, 2.00) | 0.422 | 1.16  (0.82, 1.65) | 0.390 |
| High SES: having depressed mood | 1.36  (0.53, 3.46) | 0.520 | Inf  (0, Inf) | 0.999 |
| Low SES: having perceived anxiety/depression | 1.01  (0.69, 1.49) | 0.951 | 1.00  (0.74, 1.35) | 0.998 |
| High SES: having perceived anxiety/depression | 1.35  (0.68, 2.67) | 0.389 | 0.89  (0.29, 2.72) | 0.845 |
| Low SES: ever smoker | 0.61  (0.36, 1.03) | 0.066 | 0.86  (0.54, 1.37) | 0.523 |
| High SES: ever smoker | 1.19  (0.79, 1.81) | 0.404 | Inf  (0, Inf) | 0.999 |
| Low SES: physical activity at least one day per week | 1.14  (0.84, 1.55) | 0.410 | 1.15  (0.85, 1.56) | 0.349 |
| High SES: physical activity at least one day per week | 0.98  (0.68, 1.41) | 0.911 | 2.25  (0.74, 6.83) | 0.152 |
| **Measure of effect modification on additive scale** | | | | |
|  | Men | | Women | |
|  | RERI | P-value | RERI | P-value |
| Low SES: having depressed mood | -0.31  (-0.84, 0.22) | 0.256 | -0.22  (-1.26, 0.82) | 0.680 |
| High SES: having depressed mood | -0.42  (-0.83, -0.01) | 0.044* | -1.30  (-2.14, -0.46) | 0.002** |
| Low SES: having perceived anxiety/depression | -0.04  (-0.78, 0.71) | 0.923 | 0.13  (-1.15, 1.40) | 0.844 |
| High SES: having perceived anxiety/depression | -0.50  (-0.88, -0.11) | 0.011* | 0.09  (-0.40, 0.58) | 0.720 |
| Low SES: ever smoker | 0.57  (-0.28, 1.42) | 0.190 | 0.50  (-0.01, 2.01) | 0.519 |
| High SES: ever smoker | -0.29  (-0.59, 0.00) | 0.052 | -1.15  (-2.12, -0.17) | 0.021* |
| Low SES: physical activity at least one day per week | -0.16  (-0.57, 0.25) | 0.446 | -0.25  (-0.99, 0.49) | 0.501 |
| High SES: physical activity at least one day per week | 0.04  (-0.19, 0.26) | 0.748 | -0.69  (-1.23, -0.16) | 0.011* |

***** p < .05. ** p < .01. ***p < .001.

Note: The model was adjusted for age, residence, marital status, obesity, previous diabetes mellitus, and previous hypertension. The measure of effect modification on the multiplicative scale is expressed as a Time Ratio (TR), derived by exponentiating the regression coefficients. The measure of effect modification on the additive scale was assessed using the relative excess risk due to interaction (RERI), calculated by converting TR into hazard ratios (HR) based on the parameter scale, and interpreting HR as an approximation of risk ratios (RR). Confidence intervals were estimated using the delta method.
