# Supplementary material for: Association between socioeconomic status and cardiovascular disease by sex: Mediating roles of psychological and behavioral factors
Source: PLoS One. 2026 Apr 1;21(4):e0345573. doi: 10.1371/journal.pone.0345573 (PMC13042698; doi:10.1371/journal.pone.0345573)
Supplement: S4 Table — aNo confounder–outcome risk ratio exists for the given confounder prevalence and confounder–mediator risk ratio. (DOCX) [file pone.0345573.s012.docx]

**S4 Table. Sensitivity analysis for unmeasured confounder in low socioeconomic status women with depressed mood as a mediator.**

| **Prevalence of confounder** | **Confounder-mediator risk ratio** | **Maximum confounder-outcome risk ratio** | **Critical confounder-outcome risk ratio** |
| --- | --- | --- | --- |
| 0.05 | 2.0 | 33.8 | -^a^ |
|  | 5.0 | 9.3 | -^a^ |
|  | 10.0 | 5.2 | -^a^ |
|  | 13.0 | 4.5 | -^a^ |
| 0.20 | 2.0 | 18.3 | -^a^ |
|  | 5.0 | 5.1 | -^a^ |
|  | 10.0 | 3.6 | -^a^ |
|  | 20.0 | 3.1 | -^a^ |
| 0.40 | 2.0 | 29.6 | -^a^ |
|  | 5.0 | 5.9 | -^a^ |
|  | 10.0 | 4.5 | -^a^ |
|  | 20.0 | 4.0 | -^a^ |
| 0.60 | 2.0 | -^a^ | -^a^ |
|  | 5.0 | 14.5 | -^a^ |
|  | 10.0 | 9.3 | -^a^ |
|  | 20.0 | 7.8 | -^a^ |
| 0.80 | 2.0 | -^a^ | -^a^ |
| 0.95 | 2.0 | -^a^ | -^a^ |

^a^No confounder–outcome risk ratio exists for the given confounder prevalence and confounder–mediator risk ratio.
