# Supplementary material for: Association between socioeconomic status and cardiovascular disease by sex: Mediating roles of psychological and behavioral factors
Source: PLoS One. 2026 Apr 1;21(4):e0345573. doi: 10.1371/journal.pone.0345573 (PMC13042698; doi:10.1371/journal.pone.0345573)
Supplement: S5 Table — * p < .05. ** p < .01. ***p < .001. Abbreviation: PM, proportion mediated; CI, confidence interval. Note: The model was adjusted by age, residence, marital status, obesity, previous diabetes mellitus, and previous hypertension. Income was categorized as low (1st quintile), middle (2nd–4th quintiles), and high (5th quintile). (DOCX) [file pone.0345573.s013.docx]

**S5 Table. Adjusted direct and indirect associations of income as an individual socioeconomic status indicator with cardiovascular disease via potential mediators.**

| Mediator | Income | Natural direct effect | | Natural indirect effect | | Total effect | | PM |
| --- | --- | --- | --- | --- | --- | --- | --- | --- |
|  |  | Estimate | 95% CI | Estimate | 95% CI | Estimate | 95% CI |  |
| **Men** |  |  |  |  |  |  |  |  |
| Depressed mood | | | | | | | | |
|  | Low | 1.01 | 0.86, 1.18 | 1.00 | 0.99, 1.01 | 1.01 | 0.86, 1.18 | -12.7% |
|  | Medium | 1.00 (ref) |  | 1.00 (ref) |  | 1.00 (ref) |  |  |
|  | High | 1.02 | 0.88, 1.18 | 1.00 | 1.00, 1.00 | 1.02 | 0.88, 1.18 | 2.7% |
| Perceived anxiety/depression | | | | | | | | |
|  | Low | 1.01 | 0.87, 1.19 | 0.99 | 0.99, 1.00 | 1.01 | 0.86, 1.18 | -79.7% |
|  | Medium | 1.00 (ref) |  | 1.00 (ref) |  | 1.00 (ref) |  |  |
|  | High | 1.02 | 0.88, 1.18 | 1.00 | 1.00, 1.01 | 1.02 | 0.88, 1.18 | 11.8% |
| Smoking status | | | | | | | | |
|  | Low | 1.01 | 0.86, 1.18 | 1.00 | 0.99, 1.00 | 1.01 | 0.86, 1.18 | -67.8% |
|  | Medium | 1.00 (ref) |  | 1.00 (ref) |  | 1.00 (ref) |  |  |
|  | High | 1.02 | 0.88, 1.18 | 1.00 | 0.99, 1.01 | 1.02 | 0.88, 1.18 | 3.5% |
| Physical activity | | | | | | | | |
|  | Low | 1.03 | 0.88, 1.21 | 0.98 | 0.96, 1.00 | 1.01 | 0.86, 1.18 | -235.5% |
|  | Medium | 1.00 (ref) |  | 1.00 (ref) |  | 1.00 (ref) |  |  |
|  | High | 1.02 | 0.88, 1.18 | 1.00 | 1.00, 1.01 | 1.02 | 0.88, 1.18 | 17.6% |
| **Women** |  |  |  |  |  |  |  |  |
| Depressed mood | | | | | | | | |
|  | Low | 0.85 * | 0.74, 0.99 | 0.99 * | 0.98, 0.999 | 0.84 * | 0.73, 0.98 | 6.9% |
|  | Medium | 1.00 (ref) |  | 1.00 (ref) |  | 1.00 (ref) |  |  |
|  | High | 0.96 | 0.80, 1.15 | 1.01 | 1.00, 1.01 | 0.96 | 0.80, 1.16 | -15.4% |
| Perceived anxiety/depression | | | | | | | | |
|  | Low | 0.85 * | 0.74, 0.99 | 0.99 * | 0.98, 0.996 | 0.84 * | 0.73, 0.98 | 8.0% |
|  | Medium | 1.00 (ref) |  | 1.00 (ref) |  | 1.00 (ref) |  |  |
|  | High | 0.96 | 0.80, 1.16 | 1.00 | 1.00, 1.01 | 0.96 | 0.80, 1.16 | -2.4% |
| Smoking status | | | | | | | | |
|  | Low | 0.84* | 0.73, 0.98 | 1.00 | 0.99, 1.00 | 0.84 * | 0.72, 0.98 | 2.1% |
|  | Medium | 1.00 (ref) |  | 1.00 (ref) |  | 1.00 (ref) |  |  |
|  | High | 0.96 | 0.80, 1.16 | 1.00 | 1.00, 1.01 | 0.97 | 0.80, 1.16 | -9.0% |
| Physical activity | | | | | | | | |
|  | Low | 0.84* | 0.73, 0.98 | 0.99 | 0.98, 1.00 | 0.84 * | 0.72, 0.97 | 3.6% |
|  | Medium | 1.00 (ref) |  | 1.00 (ref) |  | 1.00 (ref) |  |  |
|  | High | 0.97 | 0.81, 1.16 | 1.00 | 0.99, 1.01 | 0.97 | 0.81, 1.17 | -7.9% |

***** p < .05. ** p < .01. ***p < .001.

Abbreviation: PM, proportion mediated; CI, confidence interval.

Note: The model was adjusted by age, residence, marital status, obesity, previous diabetes mellitus, and previous hypertension. Income was categorized as low (1st quintile), middle (2nd–4th quintiles), and high (5th quintile).
