# Supplementary material for: Association between socioeconomic status and cardiovascular disease by sex: Mediating roles of psychological and behavioral factors
Source: PLoS One. 2026 Apr 1;21(4):e0345573. doi: 10.1371/journal.pone.0345573 (PMC13042698; doi:10.1371/journal.pone.0345573)
Supplement: S6 Table — * p < .05. ** p < .01. ***p < .001. Abbreviation: PM, proportion mediated; CI, confidence interval. Note: The model was adjusted by age, residence, marital status, obesity, previous diabetes mellitus, and previous hypertension. (DOCX) [file pone.0345573.s014.docx]

**S6 Table. Adjusted direct and indirect associations of working status as an individual socioeconomic status indicator with cardiovascular disease via potential mediators.**

| Mediator | Work status | Natural direct effect | | Natural indirect effect | | Total effect | | PM |
| --- | --- | --- | --- | --- | --- | --- | --- | --- |
|  |  | Estimate | 95% CI | Estimate | 95% CI | Estimate | 95% CI |  |
| **Men** |  |  |  |  |  |  |  |  |
| Depressed mood | | | | | | | | |
|  | None | 0.84* | 0.72, 0.98 | 1.00 | 0.99, 1.01 | 0.84* | 0.72, 0.98 | 0.5% |
|  | Part-time | 1.00 (ref) |  | 1.00 (ref) |  | 1.00 (ref) |  |  |
|  | Full-time | 1.03 | 0.87, 1.23 | 1.00 | 0.99, 1.01 | 1.03 | 0.87, 1.22 | -5.6% |
| Perceived anxiety/depression | | | | | | | | |
|  | None | 0.85* | 0.73, 0.999 | 0.99* | 0.98, 0.999 | 0.84* | 0.72, 0.99 | 5.5% |
|  | Part-time | 1.00 (ref) |  | 1.00 (ref) |  | 1.00 (ref) |  |  |
|  | Full-time | 1.03 | 0.87, 1.22 | 1.00 | 1.00, 1.00 | 1.03 | 0.87, 1.22 | 2.8% |
| Smoking status | | | | | | | | |
|  | None | 0.85* | 0.73, 0.99 | 1.00 | 0.99, 1.00 | 0.85* | 0.73, 0.99 | 1.1% |
|  | Part-time | 1.00 (ref) |  | 1.00 (ref) |  | 1.00 (ref) |  |  |
|  | Full-time | 1.03 | 0.87, 1.22 | 1.00 | 0.99, 1.01 | 1.03 | 0.87, 1.22 | 2.1% |
| Physical activity | | | | | | | | |
|  | None | 0.85* | 0.73, 0.996 | 1.00 | 0.99, 1.01 | 0.85* | 0.73, 0.996 | -0.5% |
|  | Part-time | 1.00 (ref) |  | 1.00 (ref) |  | 1.00 (ref) |  |  |
|  | Full-time | 1.03 | 0.87, 1.23 | 1.00 | 0.98, 1.01 | 1.03 | 0.87, 1.22 | -14.2% |
| **Women** |  |  |  |  |  |  |  |  |
| Depressed mood | | | | | | | | |
|  | None | 0.92 | 0.76, 1.11 | 1.00 | 1.00, 1.00 | 0.92 | 0.76, 1.11 | 0.8% |
|  | Part-time | 1.00 (ref) |  | 1.00 (ref) |  | 1.00 (ref) |  |  |
|  | Full-time | 0.91 | 0.65, 1.27 | 1.00 | 1.00, 1.01 | 0.91 | 0.65, 1.27 | -1.4% |
| Perceived anxiety/depression | | | | | | | | |
|  | None | 0.91 | 0.76, 1.11 | 1.00 | 0.99, 1.01 | 0.91 | 0.76, 1.11 | -0.4% |
|  | Part-time | 1.00 (ref) |  | 1.00 (ref) |  | 1.00 (ref) |  |  |
|  | Full-time | 0.91 | 0.65, 1.27 | 1.00 | 1.00, 1.00 | 0.91 | 0.66, 1.27 | -0.6% |
| Smoking status | | | | | | | | |
|  | None | 0.91 | 0.76, 1.11 | 1.00 | 1.00, 1.00 | 0.91 | 0.76, 1.11 | -0.8% |
|  | Part-time | 1.00 (ref) |  | 1.00 (ref) |  | 1.00 (ref) |  |  |
|  | Full-time | 0.90 | 0.65, 1.26 | 1.01 | 1.00, 1.01 | 0.91 | 0.65, 1.27 | -5.8% |
| Physical activity | | | | | | | | |
|  | None | 0.92 | 0.76, 1.11 | 1.00 | 0.99, 1.00 | 0.92 | 0.76, 1.11 | 2.8% |
|  | Part-time | 1.00 (ref) |  | 1.00 (ref) |  | 1.00 (ref) |  |  |
|  | Full-time | 0.92 | 0.66, 1.28 | 1.00 | 0.99, 1.01 | 0.92 | 0.66, 1.28 | 2.1% |

***** p < .05. ** p < .01. ***p < .001.

Abbreviation: PM, proportion mediated; CI, confidence interval.

Note: The model was adjusted by age, residence, marital status, obesity, previous diabetes mellitus, and previous hypertension.
