# Supplementary material for: Association between socioeconomic status and cardiovascular disease by sex: Mediating roles of psychological and behavioral factors
Source: PLoS One. 2026 Apr 1;21(4):e0345573. doi: 10.1371/journal.pone.0345573 (PMC13042698; doi:10.1371/journal.pone.0345573)
Supplement: S7 Table — * p < .05. ** p < .01. ***p < .001. Abbreviation: PM, proportion mediated; CI, confidence interval. Note: The model was adjusted by age, residence, marital status, obesity, previous diabetes mellitus, and previous hypertension. (DOCX) [file pone.0345573.s015.docx]

**S7 Table. Adjusted direct and indirect associations of education as an individual socioeconomic status indicator with cardiovascular disease via potential mediators.**

| Mediator | Education | Natural direct effect | | Natural indirect effect | | Total effect | | PM |
| --- | --- | --- | --- | --- | --- | --- | --- | --- |
|  |  | Estimate | 95% CI | Estimate | 95% CI | Estimate | 95% CI |  |
| **Men** |  |  |  |  |  |  |  |  |
| Depressed mood | | | | | | | | |
|  | ≤ Elementary school | 0.92 | 0.80, 1.06 | 1.00 | 1.00, 1.00 | 0.92 | 0.80, 1.06 | 0.02% |
|  | Middle-high school | 1.00 (ref) |  | 1.00 (ref) |  | 1.00 (ref) |  |  |
|  | ≥ College | 0.97 | 0.83, 1.14 | 1.00 | 1.00, 1.00 | 0.97 | 0.83, 1.14 | -2.8% |
| Perceived anxiety/depression | | | | | | | | |
|  | ≤ Elementary school | 0.92 | 0.80, 1.06 | 1.00 | 0.99, 1.00 | 0.92 | 0.80, 1.06 | 1.5% |
|  | Middle-high school | 1.00 (ref) |  | 1.00 (ref) |  | 1.00 (ref) |  |  |
|  | ≥ College | 0.98 | 0.83, 1.14 | 1.00 | 1.00, 1.00 | 0.97 | 0.83, 1.14 | 5.1% |
| Smoking status | | | | | | | | |
|  | ≤ Elementary school | 0.92 | 0.80, 1.06 | 1.00 | 0.99, 1.00 | 0.92 | 0.80, 1.06 | 1.7% |
|  | Middle-high school | 1.00 (ref) |  | 1.00 (ref) |  | 1.00 (ref) |  |  |
|  | ≥ College | 0.97 | 0.83, 1.13 | 1.01 | 0.99, 1.02 | 0.98 | 0.84, 1.14 | -20.8% |
| Physical activity | | | | | | | | |
|  | ≤ Elementary school | 0.93 | 0.81, 1.07 | 0.99 | 0.98, 1.00 | 0.92 | 0.80, 1.06 | 11.8% |
|  | Middle-high school | 1.00 (ref) |  | 1.00 (ref) |  | 1.00 (ref) |  |  |
|  | ≥ College | 0.97 | 0.83, 1.13 | 1.01 | 1.00, 1.02 | 0.98 | 0.84, 1.14 | -34.2% |
| **Women** |  |  |  |  |  |  |  |  |
| Depressed mood | | | | | | | | |
|  | ≤ Elementary school | 0.89 | 0.76, 1.05 | 0.99 | 0.98, 1.00 | 0.88 | 0.75, 1.04 | 5.9% |
|  | Middle-high school | 1.00 (ref) |  | 1.00 (ref) |  | 1.00 (ref) |  |  |
|  | ≥ College | 1.32 | 0.96, 1.82 | 1.00 | 1.00, 1.01 | 1.32 | 0.96, 1.82 | 1.5% |
| Perceived anxiety/depression | | | | | | | | |
|  | ≤ Elementary school | 0.89 | 0.76, 1.05 | 0.99 | 0.99, 1.00 | 0.89 | 0.75, 1.04 | 5.3% |
|  | Middle-high school | 1.00 (ref) |  | 1.00 (ref) |  | 1.00 (ref) |  |  |
|  | ≥ College | 1.32 | 0.96, 1.82 | 1.00 | 1.00, 1.00 | 1.32 | 0.96, 1.82 | 0.1% |
| Smoking status | | | | | | | | |
|  | ≤ Elementary school | 0.88 | 0.75, 1.04 | 1.00 | 0.99, 1.00 | 0.88 | 0.75, 1.04 | 2.6% |
|  | Middle-high school | 1.00 (ref) |  | 1.00 (ref) |  | 1.00 (ref) |  |  |
|  | ≥ College | 1.32 | 0.96, 1.82 | 1.00 | 0.99, 1.01 | 1.32 | 0.96, 1.82 | 0.5% |
| Physical activity | | | | | | | | |
|  | ≤ Elementary school | 0.89 | 0.76, 1.05 | 0.99 | 0.98, 1.00 | 0.88 | 0.75, 1.04 | 6.6% |
|  | Middle-high school | 1.00 (ref) |  | 1.00 (ref) |  | 1.00 (ref) |  |  |
|  | ≥ College | 1.32 | 0.96, 1.83 | 1.00 | 0.99, 1.00 | 1.32 | 0.96, 1.83 | -0.3% |

***** p < .05. ** p < .01. ***p < .001.

Abbreviation: PM, proportion mediated; CI, confidence interval.

Note: The model was adjusted by age, residence, marital status, obesity, previous diabetes mellitus, and previous hypertension.
