# Supplementary material for: Association between socioeconomic status and cardiovascular disease by sex: Mediating roles of psychological and behavioral factors
Source: PLoS One. 2026 Apr 1;21(4):e0345573. doi: 10.1371/journal.pone.0345573 (PMC13042698; doi:10.1371/journal.pone.0345573)
Supplement: S8 Table — * p < .05. ** p < .01. ***p < .001. Abbreviation: PM, proportion mediated; CI, confidence interval. Note: The model was adjusted by age, residence, marital status, obesity, previous diabetes mellitus, and previous hypertension. (DOCX) [file pone.0345573.s016.docx]

**S8 Table. Adjusted direct and indirect associations of insurance as an individual socioeconomic status indicator with cardiovascular disease via potential mediators.**

| Mediator | Insurance | Natural direct effect | | Natural indirect effect | | Total effect | | PM |
| --- | --- | --- | --- | --- | --- | --- | --- | --- |
|  |  | Estimate | 95% CI | Estimate | 95% CI | Estimate | 95% CI |  |
| **Men** |  |  |  |  |  |  |  |  |
| Depressed mood | | | | | | | | |
|  | None | Inf | 0.00, 1.00 | 0.97 | 0.83, 1.14 | Inf | 0.00, 1.00 | -2.7% |
|  | Medical aid | 1.00 (ref) |  | 1.00 (ref) |  | 1.00 (ref) |  |  |
|  | NHIS | 1.22 | 0.94, 1.58 | 1.00 | 0.96, 1.03 | 1.22 | 0.94, 1.57 | -2.2% |
| Perceived anxiety/depression | | | | | | | | |
|  | None | Inf | 0.00, 1.00 | 1.00 | 0.77, 1.30 | Inf | 0.00, 1.00 | -0.03% |
|  | Medical aid | 1.00 (ref) |  | 1.00 (ref) |  | 1.00 (ref) |  |  |
|  | NHIS | 1.18 | 0.91, 1.53 | 1.02 | 0.99, 1.05 | 1.21 | 0.93, 1.56 | 12.1% |
| Smoking status | | | | | | | | |
|  | None | Inf | 0.00, 1.00 | 1.03 | 0.64, 1.67 | Inf | 0.00, 1.00 | 3.0% |
|  | Medical aid | 1.00 (ref) |  | 1.00 (ref) |  | 1.00 (ref) |  |  |
|  | NHIS | 1.21 | 0.94, 1.57 | 1.00 | 0.99, 1.01 | 1.22 | 0.94, 1.57 | 1.7% |
| Physical activity | | | | | | | | |
|  | None | Inf | 0.00, 1.00 | 1.47 | 0.74, 2.9 | Inf | 0.00, 1.00 | 32.1% |
|  | Medical aid | 1.00 (ref) |  | 1.00 (ref) |  | 1.00 (ref) |  |  |
|  | NHIS | 1.18 | 0.91, 1.53 | 1.03 | 1.00, 1.07 | 1.22 | 0.94, 1.58 | 17.5% |
| **Women** |  |  |  |  |  |  |  |  |
| Depressed mood | | | | | | | | |
|  | None | 0.36 | 0.07, 1.89 | 1.04 | 0.93, 1.17 | 0.37 | 0.07, 1.97 | -2.4% |
|  | Medical aid | 1.00 (ref) |  | 1.00 (ref) |  | 1.00 (ref) |  |  |
|  | NHIS | 1.27 * | 1.01, 1.59 | 1.03 * | 1.00, 1.06 | 1.30 * | 1.04 1.63 | 11.7% |
| Perceived anxiety/depression | | | | | | | | |
|  | None | 0.33 | 0.06, 1.78 | 1.10 | 0.92, 1.32 | 0.36 | 0.07, 1.92 | -5.3% |
|  | Medical aid | 1.00 (ref) |  | 1.00 (ref) |  | 1.00 (ref) |  |  |
|  | NHIS | 1.24 | 0.99, 1.55 | 1.04 * | 1.02, 1.07 | 1.29 * | 1.03, 1.61 | 18.2% |
| Smoking status | | | | | | | | |
|  | None | 0.38 | 0.07, 2.02 | 0.96 | 0.83, 1.12 | 0.36 | 0.07, 1.95 | 2.1% |
|  | Medical aid | 1.00 (ref) |  | 1.00 (ref) |  | 1.00 (ref) |  |  |
|  | NHIS | 1.27 * | 1.01, 1.59 | 1.01 | 1.00, 1.02 | 1.28 * | 1.03, 1.61 | 5.2% |
| Physical activity | | | | | | | | |
|  | None | 0.39 | 0.07, 2.10 | 0.95 | 0.81, 1.12 | 0.37 | 0.07, 2.01 | 3.0% |
|  | Medical aid | 1.00 (ref) |  | 1.00 (ref) |  | 1.00 (ref) |  |  |
|  | NHIS | 1.29 * | 1.03, 1.61 | 1.01 | 0.99, 1.04 | 1.31 * | 1.04, 1.63 | 5.8% |

***** p < .05. ** p < .01. ***p < .001.

Abbreviation: PM, proportion mediated; CI, confidence interval.

Note: The model was adjusted by age, residence, marital status, obesity, previous diabetes mellitus, and previous hypertension.
