# Supplementary material for: Association between socioeconomic status and cardiovascular disease by sex: Mediating roles of psychological and behavioral factors
Source: PLoS One. 2026 Apr 1;21(4):e0345573. doi: 10.1371/journal.pone.0345573 (PMC13042698; doi:10.1371/journal.pone.0345573)
Supplement: Attachment — Submitted filename: Response to Reviewers.docx [file pone.0345573.s018.docx]

Thank you for submitting your manuscript to PLOS ONE. After careful consideration, we feel that it has merit but does not fully meet PLOS ONE’s publication criteria as it currently stands. Therefore, we invite you to submit a revised version of the manuscript that addresses the points raised during the review process.

The revised paper should address all comments.

Please submit your revised manuscript by Dec 11 2025 11:59PM. If you will need more time than this to complete your revisions, please reply to this message or contact the journal office at [plosone@plos.org](mailto:plosone@plos.org). When you're ready to submit your revision, log on to https://www.editorialmanager.com/pone/ and select the 'Submissions Needing Revision' folder to locate your manuscript file.

Please include the following items when submitting your revised manuscript:

- A rebuttal letter that responds to each point raised by the academic editor and reviewer(s). You should upload this letter as a separate file labeled 'Response to Reviewers'.
- A marked-up copy of your manuscript that highlights changes made to the original version. You should upload this as a separate file labeled 'Revised Manuscript with Track Changes'.
- An unmarked version of your revised paper without tracked changes. You should upload this as a separate file labeled 'Manuscript'.

If you would like to make changes to your financial disclosure, please include your updated statement in your cover letter. Guidelines for resubmitting your figure files are available below the reviewer comments at the end of this letter.

If applicable, we recommend that you deposit your laboratory protocols in protocols.io to enhance the reproducibility of your results. Protocols.io assigns your protocol its own identifier (DOI) so that it can be cited independently in the future. For instructions see: <https://journals.plos.org/plosone/s/submission-guidelines#loc-laboratory-protocols>. Additionally, PLOS ONE offers an option for publishing peer-reviewed Lab Protocol articles, which describe protocols hosted on protocols.io. Read more information on sharing protocols at <https://plos.org/protocols?utm_medium=editorial-email&utm_source=authorletters&utm_campaign=protocols>.

**Journal Requirements:**

When submitting your revision, we need you to address these additional requirements.

1. Please ensure that your manuscript meets PLOS ONE's style requirements, including those for file naming. The PLOS ONE style templates can be found at

<https://journals.plos.org/plosone/s/file?id=wjVg/PLOSOne_formatting_sample_main_body.pdf>

<https://journals.plos.org/plosone/s/file?id=ba62/PLOSOne_formatting_sample_title_authors_affiliations.pdf>

We have completed the revisions according to PLOS ONE's style requirements.

2. We note that you have indicated that there are restrictions to data sharing for this study. For studies involving human research participant data or other sensitive data, we encourage authors to share de-identified or anonymized data. However, when data cannot be publicly shared for ethical reasons, we allow authors to make their data sets available upon request. For information on unacceptable data access restrictions, see

<http://journals.plos.org/plosone/s/data-availability#loc-unacceptable-data-access-restrictions>

Before we proceed with your manuscript, please address the following prompts:

a) If there are ethical or legal restrictions on sharing a de-identified data set, please explain them in detail (e.g., data contain potentially identifying or sensitive patient information, data are owned by a third-party organization, etc.) and who has imposed them (e.g., a Research Ethics Committee or Institutional Review Board, etc.). Please also provide contact information for a data access committee, ethics committee, or other institutional body to which data requests may be sent.

 We contacted the data-providing institutions, The Korea Institute for Health and Social Affairs (KIHASA) and the National Health Insurance Service, and received the following response:

"If the materials are intended to be attached for journal publication, please indicate the data source rather than submitting the dataset itself.

All annual datasets from Panel 1 and Panel 2 of the Korea Health Panel Survey can be downloaded from the Korea Health Panel Survey website (https://www.khp.re.kr:444/).

(For data download: <https://www.khp.re.kr:444/web/data/board/dataDownload.do?bbsid=107> )

If the data are being requested solely for manuscript review, it is permissible to submit processed data created by the researcher, containing only the minimum information necessary and focused on the variables used in the study.

Therefore, while it may be possible to provide a minimal processed dataset during the journal review process, uploading the data to a public data repository is likely to be difficult.”

We are also sharing the institution’s contact information for further inquiries: [khp@kihasa.re.kr](mailto:khp@kihasa.re.kr)

b) If there are no restrictions, please upload the minimal anonymized data set necessary to replicate your study findings to a stable, public repository and provide us with the relevant URLs, DOIs, or accession numbers. Please see <http://www.bmj.com/content/340/bmj.c181.long> for guidelines on how to de-identify and prepare clinical data for publication. For a list of recommended repositories, please see https://journals.plos.org/plosone/s/recommended-repositories. You also have the option of uploading the data as Supporting Information files, but we would recommend depositing data directly to a data repository if possible.

Because there were constraints on uploading the data to the recommended repositories, we instead uploaded the processed files as supplementary materials for the review process (file name: data.csv).

Please update your Data Availability statement in the submission form accordingly.

We described the data availability statement as follows:

The data underlying this study belong to the Korea Institute for Health and Social Affairs (KIHASA). Researchers interested in the data may download them from the KIHASA website (<https://www.khp.re.kr:444/web/data/board/dataDownload.do?bbsid=107>) or contact the Korea Health Panel Study (email: [khp@kihasa.re.kr](mailto:khp@kihasa.re.kr)).

3. Thank you for uploading your study's underlying data set. Unfortunately, the repository you have noted in your Data Availability statement does not qualify as an acceptable data repository according to PLOS's standards.

At this time, please upload the minimal data set necessary to replicate your study's findings to a stable, public repository (such as figshare or Dryad) and provide us with the relevant URLs, DOIs, or accession numbers that may be used to access these data. For a list of recommended repositories and additional information on PLOS standards for data deposition, please see <https://journals.plos.org/plosone/s/recommended-repositories>.

 We apologize, but although we intended to upload the data to an appropriate repository, we were unable to do so in accordance with the recommendations of the data-providing institution. Instead, we uploaded the processed data files to the additional files (file name: data.csv).

4. If the reviewer comments include a recommendation to cite specific previously published works, please review and evaluate these publications to determine whether they are relevant and should be cited. There is no requirement to cite these works unless the editor has indicated otherwise.

We were asked to add a reference regarding the method of data collection, and we cited a paper that describes the dataset used.

Jin, D. L., Go, D. S., & Yoon, S. J. (2025). Longitudinal perspectives on health and medical research in Korea: strengths and limitations of key panel datasets. Journal of Korean Medical Science, 40(23).

**Additional Editor Comments:**

The revised paper should address all comments.


[Note: HTML markup is below. Please do not edit.]

**Reviewers' comments:**

Reviewer's Responses to Questions

**Comments to the Author**

1. Is the manuscript technically sound, and do the data support the conclusions?

The manuscript must describe a technically sound piece of scientific research with data that supports the conclusions. Experiments must have been conducted rigorously, with appropriate controls, replication, and sample sizes. The conclusions must be drawn appropriately based on the data presented.

Reviewer #1: Yes

Reviewer #2: Partly

Reviewer #3: Yes

2. Has the statistical analysis been performed appropriately and rigorously?

Reviewer #1: Yes

Reviewer #2: I Don't Know

Reviewer #3: Yes

3. Have the authors made all data underlying the findings in their manuscript fully available?

The [PLOS Data policy](http://www.plosone.org/static/policies.action#sharing) requires authors to make all data underlying the findings described in their manuscript fully available without restriction, with rare exception (please refer to the Data Availability Statement in the manuscript PDF file). The data should be provided as part of the manuscript or its supporting information, or deposited to a public repository. For example, in addition to summary statistics, the data points behind means, medians and variance measures should be available. If there are restrictions on publicly sharing data—e.g. participant privacy or use of data from a third party—those must be specified.

Reviewer #1: Yes

Reviewer #2: No

Reviewer #3: No

4. Is the manuscript presented in an intelligible fashion and written in standard English?

PLOS ONE does not copyedit accepted manuscripts, so the language in submitted articles must be clear, correct, and unambiguous. Any typographical or grammatical errors should be corrected at revision, so please note any specific errors here.

Reviewer #1: Yes

Reviewer #2: No

Reviewer #3: Yes

5. Review Comments to the Author

Please use the space provided to explain your answers to the questions above. You may also include additional comments for the author, including concerns about dual publication, research ethics, or publication ethics. (Please upload your review as an attachment if it exceeds 20,000 characters)

**Reviewer #1:** The study is methodologically solid and well executed. Minor improvements such as consistent abbreviation use, correcting typographical errors, and expanding policy recommendations; would further strengthen it.

Overall, it offers valuable insights into socioeconomic inequalities in cardiovascular health.

We deeply appreciate to carefully reading our text. We have now carefully considered the reviewer’s comments and reflected them in the revised manuscript to improve clarity and precision.

● In the revised manuscript, we have ensured that all abbreviations, including socioeconomic status (SES) and total effect (TE), are clearly spelled out at their first appearance in each section for clarity and readability.

[Page 2 Lines 12-14] Previous research shows that low socioeconomic status (SES) increases the risk of cardiovascular disease (CVD) and contributes to health disparities through the unequal distribution of intermediary factors.

[Page 16 Lines 238-243] In women, low SES on average had a 17% shorter survival time until their first CVD compared to medium SES, and 2% could be attributed to depressed mood (NDE 0.83, 95% confidence interval (CI) 0.72-0.97; NIE 0.98, 95% CI 0.97-0.999; Total effect (TE) 0.82, 95% CI 0.71-0.95) and perceived anxiety/depression (NDE 0.84, 95% CI 0.73-0.97; NIE 0.98, 95% CI 0.97-0.99; TE 0.83, 95% CI 0.72-0.96).

[Page 18 Line 247] Abbreviation: SES, socioeconomic status; PM, proportion mediated; CI, confidence interval.

● We have corrected typographical errors and grammatically reviewed the entire text.

● We have added specific policy recommendations to the discussion section.

[Page 22 Lines 343-349] Therefore, policies such as community‑based mental health screening and accessible treatment services for low‑SES women should be implemented to reduce CVD risk. Targeted interventions should prioritize women in low‑SES groups by integrating subsidized mental health services into primary care. In addition, coordinated policies that combine socioeconomic support with accessible psychological care for low‑SES women can reduce health inequities and ultimately lower CVD risk. Our findings underscore the broader importance of integrating mental health into cardiovascular prevention strategies to reduce CVD risk among low‑SES women.

**Reviewer #2:** It seems that the study conducted is not well described and has many ambiguities.

We appreciate you taking the time to read our text attentively. We have carefully taken into account the reviewer's comments and included them in the revised manuscript to improve the novelty of our study.

• The type of the current study is not stated.

Thank you for the valuable comment. We have added the study type to the Materials and Methods section.

[Page 6 Lines 93-95] This retrospective cohort study utilized the Korea Health Panel Survey (KHPS) data (version 1.7.3.), which was conducted annually from 2008 to 2018 by the Korea Institute for Health and Social Affairs and the National Health Insurance Service (NHIS) consortium.

• The data related to the years 2008 to 2018, which may have resulted in the loss of valuable data due to rapid global changes.

We appreciate the reviewer’s insightful comment regarding the study period. Unfortunately, the available dataset only covered the years 2008 to 2018, and therefore we were unable to include more recent data in our analysis. We acknowledge that rapid global changes after 2018 may influence the outcomes; however, our study was designed to utilize the most comprehensive data accessible at the time. Despite this limitation, the large sample size and robust methodology provide meaningful and reliable findings that contribute to the understanding of the research question. We have added a statement in the limitations section to clarify this point.

[Page 22 Lines 325-327] Second, the available dataset only covered the years 2008 to 2018, which may not fully reflect more recent socioeconomic and cardiovascular changes.

• Also, the introduction of the study cannot fully explain the necessity and importance of the study.

Thank you for your precious comment. To emphasize the significance of this study, we revised the title and thoroughly rewrote the introduction as follows:

Title: Association between socioeconomic status and cardiovascular disease by sex: mediating roles of psychological and behavioral factors

- The first paragraph presents the current status of cardiovascular disease (CVD).
- The second paragraph discusses CVD risk factors, highlights the association with socioeconomic status (SES), and introduces the conceptual framework of mediating factors.
- The third paragraph examines the mechanisms of psychological factors such as depression and anxiety in the relationship between low SES and CVD.
- The fourth paragraph addresses multifaceted social factors and health behaviors as mediating mechanisms, explaining smoking and physical activity as pathways to CVD.
- The fifth paragraph reviews previous studies on mediators and identifies their limitations.
- The sixth paragraph points out the shortcomings of using single SES indicators and emphasizes the need for composite measures, while the seventh paragraph provides examples of SES research employing latent class analysis (LCA).
- The final paragraph highlights the gaps in the existing literature and states the aim of the present study.

• The research method section seems to have many shortcomings. The method of data collection should be fully explained and in some cases requires references.

We provided a more detailed description of data collection in the manuscript and additionally included a reference to a study that explains data collection from the Korea Health Panel.

[Page 6 Lines 95-101] This panel employs a multi-stage stratified probability sampling method using 90% of the 2005 Population and Housing Census data across sixteen districts nationwide to maintain national representativeness. KHPS collected variables through face-to-face interviews using the Computer Assisted Personal Interviewing (CAPI) method [29]. Although the data were self-reported, their reliability was enhanced by the panel households’ completion of a one-year health diary on medical utilization and the collection of medical receipts and year-end tax settlement records.

[Page 7 Lines 124-127] The main outcome was newly diagnosed CVD cases, assessed through self‑reported physician diagnoses. Participants reported the physician’s diagnosis name and the corresponding International Classification of Diseases 10th edition (ICD-10) code during surveys of emergency, inpatient, and outpatient service use.

*reference: Jin, D. L., Go, D. S., & Yoon, S. J. (2025). Longitudinal perspectives on health and medical research in Korea: strengths and limitations of key panel datasets. Journal of Korean Medical Science, 40(23).

We also supplemented the statistical explanation of the mediation analysis.

[Page 9 Lines 167-177] Causal mediation analysis provides a more general and rigorous framework for survival models, accommodating nonlinear relationships and interactions [34]. It needs assumptions for valid estimation of natural direct effects (NDE) and natural indirect effects (NIE): measured covariates must control for confounding in the (1) exposure–outcome, (2) mediator–outcome, and (3) exposure–mediator relationships, and (4) mediator–outcome confounders must not be influenced by the exposure [35]. The accelerated failure time (AFT) model was formulated using survival functions, with the Weibull distribution showing the best fit based on AIC criteria (S2 Table). The AFT model represents time ratios, interpreted as a reduction (less than 1) for deleterious covariates and an increase (greater than 1) for protective covariates. The proportion mediated (PM) was calculated following the method proposed by VanderWeele and Vansteelandt [36]. Mediation analysis was conducted using a SAS macro that incorporates causal effects estimated on the mean survival ratio scale within the AFT model [37].

• How was the health behavior variable assessed? This variable has a measurable scale. But how was it collected in this study?

In Korea, health behavior scales include measures such as alcohol consumption, stress and mental health, physical activity, etc (Shin, 2010). However, previous meta-analyses have shown that alcohol consumption of approximately 2.5–14.9 g/day (about ≤1 drink per day) may be associated with a reduced risk of CVD (Ronksley et al, 2011). Therefore, examining a composite scale that includes alcohol intake may not be appropriate when evaluating its association with CVD; rather, it may be more suitable to assess health behaviors as independent indicators.

Unfortunately, the survey instrument used to collect data in the present study does not include a health behavior scale, making it difficult to adopt such a scale directly. Instead, based on a review of the existing literature, we identified behavioral factors that mediate the relationship between SES and CVD. We found that in studies examining the association between socioeconomic position and mortality from cardiovascular diseases, behavioral mediators were defined as smoking, alcohol consumption, physical inactivity, poor diet, and body mass index (Hossin et al, 2021). Therefore, in this study, health behaviors were defined using two variables: physical activity and smoking status.

*references:

Shin, Y. H. (2010). Development and psychometric evaluation of a scale to measure health behaviors of adolescents. Journal of Korean Academy of Nursing, 40(6), 820-830.

Ronksley, P. E., Brien, S. E., Turner, B. J., Mukamal, K. J., & Ghali, W. A. (2011). Association of alcohol consumption with selected cardiovascular disease outcomes: a systematic review and meta-analysis. Bmj, 342, d671.

Hossin, M. Z., Koupil, I., & Falkstedt, D. (2021). Early life socioeconomic position and mortality from cardiovascular diseases: an application of causal mediation analysis in the Stockholm Public Health Cohort. BMJ open, 9(6), e026258.

• Also, the study discussion and conclusion section is poorly written. What are the implications of the present results for health promotion in society? What are the basic and practical suggestions for health policymakers?

We appreciate the reviewer’s comment regarding the discussion and conclusion section. We have added concrete recommendations, including community‑based screening, integration of subsidized mental health services into primary care, and coordinated socioeconomic support policies for low‑SES women.

[Page 22 Lines 343-349] Therefore, policies such as community‑based mental health screening and accessible treatment services for low‑SES women should be implemented to reduce CVD risk. Targeted interventions should prioritize women in low‑SES groups by integrating subsidized mental health services into primary care. In addition, coordinated policies that combine socioeconomic support with accessible psychological care for low‑SES women can reduce health inequities and ultimately lower CVD risk. Our findings underscore the broader importance of integrating mental health into cardiovascular prevention strategies to reduce CVD risk among low‑SES women.

• Literary review supports the study variables, especially the mediating variables, in terms of their effect on the main dependent variable, which is cardiovascular disease, not being well expressed.

We appreciate the reviewer’s comment regarding the insufficient expression of mediating variables in the literature review. In revision, we have expanded the introduction to emphasize how psychological factors (depressed mood, perceived anxiety/depression) and behavioral factors (smoking, physical activity) contribute to CVD. We now highlight evidence showing that low SES is linked to higher rates of depression and perceived anxiety/depression, which elevate CVD risk through biological stress pathways, while smoking and physical inactivity further increase risk as key modifiable behaviors. We also incorporated findings from prior studies quantifying these mediating effects and noted their limitations.

[Page 3 Lines 45 - Page 4 Lines 68] Low income is associated with having depression and anxiety, both of which represent established independent risk factors for CVD [9, 10]. Meta-analyses have shown that low income is associated with increased depression [11]. Individuals with depression may experience dysregulation of the sympathetic nervous system and the hypothalamic–pituitary–adrenal axis, leading to coronary vasoconstriction, endothelial dysfunction, and increased platelet activation, ultimately elevating cardiac risk [10]. Structured diagnostic interviews suggest that lower SES is associated with a higher likelihood of anxiety [12]. Stress and anxiety can promote atherosclerosis and may serve as acute triggers of major cardiac events, thereby increasing CVD risk [13, 14].

Health behaviors are recognized as key mediating mechanisms linking distal structural factors to individual health outcomes [15]. Health behaviors are shaped by multifaceted social, economic, and environmental factors and they exhibit strong social patterning [16]. Smoking is a major risk factor for CVD, and individuals who smoke have a significantly higher risk of CVD compared to non-smokers [17]. Sedentary behavior and physical activity are modifiable risk factors for CVD, with sedentary behavior increasing CVD risk and higher levels of physical activity reducing CVD risk [18]. These results can be explained by physical activity improving lipid profiles—raising HDL and lowering triglycerides and total cholesterol—which in turn reduces CVD risk.

Previous studies have explored psychological and behavioral mediators in the SES–CVD association. Depression was reported to account for 10.9% of this relationship, although the cross-sectional design limited causal inference [19]. Other research identified smoking, physical inactivity, and alcohol use as mediators of the SES–ischemic heart disease mortality link [20], while cigarette smoking and physical inactivity explained 11.4% and 7.7% of the association between social determinants of health (SDOH) and CVD mortality, respectively [21]. However, the use of a composite SDOH score based on the number of unfavorable indicators makes it difficult to disentangle the specific contribution of each attribute.

• This study also has many methodological ambiguities. Did a statistician oversee the data and how the study was analyzed?

Thank you for your precious comment. We have provided a detailed description of the methods section, elaborated on causal mediation analysis, and added relevant references.

[Page 9 Lines 167-177] Causal mediation analysis provides a more general and rigorous framework for survival models, accommodating nonlinear relationships and interactions [34]. It needs assumptions for valid estimation of natural direct effects (NDE) and natural indirect effects (NIE): measured covariates must control for confounding in the (1) exposure–outcome, (2) mediator–outcome, and (3) exposure–mediator relationships, and (4) mediator–outcome confounders must not be influenced by the exposure [35]. The accelerated failure time (AFT) model was formulated using survival functions, with the Weibull distribution showing the best fit based on AIC criteria (S2 Table). The AFT model represents time ratios, interpreted as a reduction (less than 1) for deleterious covariates and an increase (greater than 1) for protective covariates. The proportion mediated (PM) was calculated following the method proposed by VanderWeele and Vansteelandt [36]. Mediation analysis was conducted using a SAS macro that incorporates causal effects estimated on the mean survival ratio scale within the AFT model [37].

• The structure of the present manuscript is not fluent and does not create interest in conveying the research results.

We thank the reviewer for the valuable comment regarding the structure of the manuscript. In response, we have reorganized the introduction and discussion to improve clarity and flow. The revised manuscript now follows a more coherent structure by focusing on psychological factors (depressed mood and perceived anxiety/depression) and health behaviors (physical activity and smoking) as mediators in the SES–CVD association. We have added more detailed explanations of the data and causal mediation analysis, and additionally performed a sensitivity analysis to assess the impact of unmeasured confounding. We also added to the discussion that the individual SES components showed different results between men and women. This restructuring allows the narrative to progress logically from background to mechanisms and finally to policy implications, thereby enhancing readability and better conveying the significance of our findings.

• It seems that if the present study had been conducted in a smaller sample population but in a more precise manner and the variables, especially the health behavior variable, had been examined with an appropriate scale, it would have had higher scientific value.

We deeply appreciate your comment. We examined the overall mediating effects by categorizing the mediators into psychological factors (depressed mood and perceived anxiety/depression) and health behaviors (physical activity and smoking). Although health behaviors were not collected as a composite scale in the survey and therefore could not be operationalized as such, we selected the variables based on established definitions in the literature.

As this is a mediation analysis, we also verified the temporal ordering of SES, mediator factors, and CVD onset. The sample size decreased due to the removal of missing values after including the perceived anxiety/depression variable. Even with the reduced sample size, we found that depressed mood and perceived anxiety/depression mediated the association in women.

**Reviewer #3:** This manuscript investigates the association between socioeconomic status (SES) and cardiovascular disease (CVD) incidence by sex, exploring mediation by health behaviors, depression, and unmet medical needs using data from the Korea Health Panel Survey (KHPS) 2009–2018. Employing latent class analysis (LCA) to derive SES and causal mediation analysis (AFT model), the authors find that low SES is significantly associated with shorter survival to CVD among women, partially mediated by depression.

We deeply appreciate to carefully reading our text. We have now carefully considered the reviewer’s comments and reflected them in the revised manuscript to improve novelty.

Here are the main points that should be addressed before publication:
• The manuscript must clearly articulate the timing of measurement for SES, mediators, and CVD incidence and discuss whether causal mediation assumptions (such as no unmeasured confounding between mediator and outcome) are plausible. Without temporal separation, the mediation interpretation becomes weaker.

We have added the measurement time points for each variable in the manuscript. Within the dataset, no cases were identified in which the year of SES measurement occurred after the year of mediator measurement, or in which the mediator measurement occurred after the year of CVD incidence. Although the sample size changed due to the exclusion of missing data after adding the perceived anxiety/depression variable, we confirmed that all individuals in the updated analytic sample continued to satisfy these conditions.

[Page 6 Lines 105-110] The study population was selected after applying the following exclusions: 430 individuals with a history of CVD before the index date; 7,335 individuals younger than 30 years at baseline; 126 individuals whose mediator factors were measured before the baseline assessment or after the date of CVD onset; and 1,239 individuals who did not complete or respond to surveys on health behaviors, income, and depressed mood (**Fig 1**).

[Page 7 Lines 129-130] We confirmed that the timing of measurements did not violate the temporal order of exposure variables, mediator variables, and CVD onset.

[Page 7 Lines 138-139] All mediator variables were measured after the baseline assessment of the exposure variable.

• How is depression measured (e.g., PHQ-9 score, clinical diagnosis)? The validity and cut‐off used need clear description.

Thank you for your valuable comment. Depression symptom (now referred to as depressed mood) were defined as responding “yes” to having felt very sad or unhappy to the extent that daily life was impaired for at least two consecutive weeks during the past year. This item is also used as a criterion for diagnosing major depression and corresponds to the “persistently low or depressed mood” criterion for major depressive disorder. As the reviewer pointed out, although neither a clinical diagnosis nor the PHQ-9 was used, the terms “depression” and “depression symptoms” had been used indiscriminately throughout the manuscript. Therefore, we revised the terminology and consistently replaced them with “depressed mood.” We also addressed in the limitations section that our definition was based on perceived feelings rather than clinical diagnoses.

***References**

Bains N, Abdijadid S. Major Depressive Disorder. [Updated 2023 Apr 10]. In: StatPearls [Internet]. Treasure Island (FL): StatPearls Publishing; 2025 Jan-. Available from: <https://www.ncbi.nlm.nih.gov/books/NBK559078/>

<https://www.nimh.nih.gov/health/publications/depression>

[page 22 lines 323-325] In addition, psychological factors were assessed through self-reported feelings rather than physician diagnoses; therefore, future mediation analyses using clinically diagnosed factors are warranted.

• Unmet medical needs: This is often self-reported and prone to recall/reporting bias. The manuscript should discuss how this variable is operationalized and its reliability.

We fully agree with the reviewer’s comment that unmet medical needs, as they are self-reported, are susceptible to recall bias and therefore difficult to operationalize. To address all reviewer comments, we excluded unmet medical needs from our variables and categorized the mediators into two domains—psychological factors (depressed mood and perceived anxiety/depression) and health behaviors (physical activity and smoking)—to examine their mediating effects accordingly.

• Cardiovascular disease (CVD): Is this a self-reported physician diagnosis, hospital record, or self-report? Are incident vs prevalent cases distinguished? Clarity is needed.

CVD was assessed as a self‑reported physician diagnosis. In the surveys on emergency service use, inpatient service use, and outpatient service use, participants were asked to report the physician’s diagnosis name and corresponding diagnostic code. Although the data were self‑reported, the reliability was enhanced because each panel household maintained a one‑year health diary of medical utilization and submitted medical receipts and year‑end tax records.

For the definition of CVD, we excluded individuals who reported ischemic heart disease (ICD‑10: I20–I25) or stroke (ICD‑10: I60–I69) in the chronic disease and medication use survey. In addition, any cases that occurred prior to the index date were removed from the emergency, inpatient, and outpatient service records. As shown in Figure 1, 430 individuals with a prior history of CVD were excluded. Therefore, only incident cases of CVD were included in the analysis, and this has been clearly described in the manuscript.

[Page 7 Lines 124-130] The main outcome was newly diagnosed CVD cases, assessed through self‑reported physician diagnoses. Participants reported the physician’s diagnosis name and the corresponding International Classification of Diseases 10th edition (ICD-10) code during surveys of emergency, inpatient, and outpatient service use. We defined CVD as ischemic heart disease (ICD-10: I20–I25) or stroke (ICD-10: I60–I69) [30]. The participants were censored by a CVD event, death, or 31 December 2018, whichever occurred first. We confirmed that the timing of measurements did not violate the temporal order of exposure variables, mediator variables, and CVD onset.

• The authors did not perform a priori power or sample-size calculation. They simply used all available respondents (n = 11 397) from the KHPS dataset without stating the detectable effect size, statistical power, or design effect.

In this study, the proportional hazards (PH) assumption was not satisfied; therefore, we employed an accelerated failure time (AFT) model. For sample size determination, we used methods that do not rely on the PH assumption rather than those proposed by Schoenfeld. Specifically, we adopted the average hazard ratio approach suggested by Dormuth et al. (2024) and applied simulation‑based sample size calculation.

Since SES was categorized into three groups, we conducted separate analyses comparing the medium and low groups, as well as the medium and high groups. A Bonferroni adjustment was applied, and thus the alpha level was divided by the number of tests, resulting in a significance level of 0.025. For the medium versus low comparison, we confirmed that with 7,390 individuals in the control group, the power reached 1.0. For the medium versus high comparison, with 7,599 individuals in the control group, the power achieved was 0.98. In our study, the medium group comprised 7,632 individuals, which exceeds the required sample size indicated by the calculations.

*Reference: Dormuth, I., Pauly, M., Rauch, G., & Herrmann, C. (2024). Sample Size Calculation Under Nonproportional Hazards Using Average Hazard Ratios. Biometrical Journal, 66(6), e202300271.

• The dataset shows approximately 79% rural residents, much higher than Korea’s real rural proportion (18%), implying sampling distortion. It needs clarification.

We confirmed from the World Bank Group that the rural population in Korea is approximately 18%. The source notes that there is no universal standard for distinguishing rural from urban areas, and that the methodology for defining rural populations is determined by each country’s national statistical office.

(<https://data.worldbank.org/indicator/SP.RUR.TOTL.ZS?locations=KR>)

However, the figures reported in the *Current Status of Urban Population* by the official Korean authority, the Ministry of Data and Statistics, differ considerably. When applying the two criteria used to define urban areas—(1) zoning classification (urban areas: residential, commercial, industrial, and green zones; non-urban areas: agricultural/forestry and environmental conservation zones) and (2) administrative district classification (urban: *eup* and *dong*; rural: *myeon*)—the proportion of urban population in 2009, the baseline year of our study, was 90.8% and 89.7%, respectively. In our study, urban areas were defined as the Seoul and Gyeonggi regions, which accounted for approximately 20% of the study population.

(<https://kosis.kr/statHtml/statHtml.do?orgId=460&tblId=TX_315_2009_H1001&conn_path=I2&language=en>)

Studies using the National Health Insurance Service (NHIS) database have also categorized Seoul, Gyeonggi, and Incheon as the Seoul metropolitan area. Accordingly, we revised our definition in the present study to classify Seoul, Gyeonggi, and Incheon as the Seoul metropolitan area. In the Chung et al study, the Seoul metropolitan area accounted for 33.6% of 33,152 participants, whereas in our study, it comprised 41.2% of 11,265 participants.

*reference: Chung, S. Y., Lee, R. G., Park, S. Y., & Oh, I. H. (2024, January). Disability-related mortality inequity in South Korea: Comparison in Terms of seoul metropolitan/non-metropolitan areas and income levels. In Healthcare (Vol. 12, No. 3, p. 293). MDPI.

• The manuscript must discuss potential confounders (e.g., comorbidities, genetic risk, baseline health status) and what has been adjusted for. If some confounders are unmeasured, this weakens the ability to interpret the mediation effects causally.

We explored previous studies that examined health behaviors and depression as mediators in the relationship between SES and CVD, and reviewed the confounders used in those studies. Accordingly, in this study we additionally adjusted for obesity, history of diabetes mellitus, and history of hypertension.

| **Confounders** | **References** |
| --- | --- |
| marital status, race and ethnicity, and survey year | Behavioral risk factors and socioeconomic inequalities in ischemic heart disease mortality in the United States: A causal mediation analysis using record linkage data |
| age; sex; marital status; self-reported race; an acculturation score based on the country of birth, length of time in the US or UK, and language spoken at home; history of hypertension, diabetes, CVD, or cancer; and history of chronic bronchitis, emphysema, or chronic obstructive pulmonary disease | Associations of healthy lifestyle and socioeconomic status with mortality and incident cardiovascular disease: two prospective cohort studies |
| baseline confounders (age and cohort) | Education and risk of coronary heart disease: assessment of mediation by behavioral risk factors using the additive hazards model |
| age, sex, race and ethnicity, marital status, educational level, body mass index, smoking status, anti-diabetic drug, anti-hypertensive drug, and anti-hyperlipidemic drug | The mediating role of depression in the association between socioeconomic status and cardiovascular disease: A nationwide cross-sectional study from |
| country of birth, age, gender and marital status | Early life socioeconomic position and mortality from cardiovascular diseases: an application of causal mediation analysis in the Stockholm Public Health Cohort |
| genetics | Depression and cardiovascular disease: epidemiological evidence on their linking mechanisms |

[Page 8 Lines 145-153] Based on previous research on potential confounders, the adjustments included were age, residence, marital status, obesity, history of diabetes mellitus, and history of hypertension [19, 27]. Age was categorized into 30-39, 40-49, 50-59, and 60 and above, while residence was classified as Seoul metropolitan for Seoul, Gyeonggi, and Incheon and Non-metropolitan for other areas [31]. Marital status was categorized as never married, married, separated/divorced, and widowed. Obesity was defined as a body mass index (BMI) of 25 or higher, with BMI calculated as weight divided by height squared. History of diabetes mellitus (ICD‑10: E10–E14) and history of hypertension (ICD‑10: I10) were defined as having the condition at baseline. However, unmeasured confounding, such as genetic factors that are difficult to measure in this study, may also exist [32].

However, genetic factors and other influences may act as unmeasured confounders in the mediator–outcome relationship. Therefore, we applied sensitivity analysis in this study.

[Page 9 Lines 182-190] Second, we conducted a sensitivity analysis to evaluate how unmeasured mediator–outcome confounding, varying in strength, prevalence, and associations, could influence the observed effects and determine the level of confounding required to alter their interpretation [39]. Combinations of confounder prevalence and confounder–mediator risk ratios (RR_CM_), together with the corresponding maximum confounder–outcome risk ratios (RR_CY_) that could be accommodated by the additive interaction model, were presented. As an example, we used the results of the indirect effects with depressed mood as a mediator among women and applied the additive model for the pure indirect effect (PIE) (S5 Table, S1-S6 Figs).

[Page 19 Lines 249-254] S5 Table presents the range of possible confounder–mediator and confounder–outcome risk ratios for the additive model in low SES women with depressed mood as a mediator. However, none of the confounding scenarios examined were consistent with a proportion explained by the adjusted PIE effect of 7.2%, indicating that the observed indirect effect is unlikely to be fully explained by unmeasured confounding. S1–S6 Figs show the impact of each confounder with a given prevalence (e.g., 5%, 20%, 40%, 60%, 80%, and 95%) in the additive model, where the PIE accounted for 2.3% of the total effect.

We have addressed the issue of potential unmeasured confounders in the limitations section of the manuscript. We performed a sensitivity analysis to address this limitation; however, we documented that time was not adequately considered, as the analysis relied solely on relative risk.

[Page 22 Lines 327-330] Third, the assumptions for causal mediation analysis require no unmeasured confounding; however, factors such as social support, genetics, and environment may serve as potential unmeasured confounders. Accordingly, we conducted a sensitivity analysis; however, because the estimates were based solely on relative risk, the consideration of time was insufficient.

• The study is based on Korean data; this should be noted in limitations regarding extrapolation to other contexts (e.g., LMICs, differing health systems, and access issues). Also, the SES latent class definitions will be culturally specific.

We appreciate the reviewer’s insightful comment. We agree that the generalizability of our findings may be limited, as the study is based on Korean data. We have now acknowledged these points in the limitations section of the manuscript.

[Page 22 Lines 330-334] Fourth, this study is based on Korean data, and caution should be exercised when extrapolating the findings to other contexts, such as low- and middle-income countries and countries with differing health systems and access to healthcare. In addition, the SES latent class definitions employed in this study are culturally specific and may not be directly generalizable to other populations.

• The use of abbreviations (e.g., SES and TE) should be clearly explained at first mention in each section.

Thank you for the helpful comment. In the revised manuscript, we have ensured that all abbreviations, including socioeconomic status (SES) and total effect (TE), are clearly spelled out at their first appearance in each section for clarity and readability.

[Page 2 Lines 12-14] Previous research shows that low socioeconomic status (SES) increases the risk of cardiovascular disease (CVD) and contributes to health disparities through the unequal distribution of intermediary factors.

[Page 16 Lines 238-243] In women, low SES on average had a 17% shorter survival time until their first CVD compared to medium SES, and 2% could be attributed to depressed mood (NDE 0.83, 95% confidence interval (CI) 0.72-0.97; NIE 0.98, 95% CI 0.97-0.999; Total effect (TE) 0.82, 95% CI 0.71-0.95) and perceived anxiety/depression (NDE 0.84, 95% CI 0.73-0.97; NIE 0.98, 95% CI 0.97-0.99; TE 0.83, 95% CI 0.72-0.96).

[Page 18 Line 247] Abbreviation: SES, socioeconomic status; PM, proportion mediated; CI, confidence interval.

6. PLOS authors have the option to publish the peer review history of their article ([what does this mean?](https://journals.plos.org/plosone/s/editorial-and-peer-review-process#loc-peer-review-history)). If published, this will include your full peer review and any attached files.
If you choose “no”, your identity will remain anonymous but your review may still be made public.
**Do you want your identity to be public for this peer review?** For information about this choice, including consent withdrawal, please see our [Privacy Policy](https://www.plos.org/privacy-policy).

Reviewer #1: **Yes:** Faisal Alkulaib	Reviewer #2: No	Reviewer #3: No

[NOTE: If reviewer comments were submitted as an attachment file, they will be attached to this email and accessible via the submission site. Please log into your account, locate the manuscript record, and check for the action link "View Attachments". If this link does not appear, there are no attachment files.]

To ensure your figures meet our technical requirements, please review our figure guidelines: <https://journals.plos.org/plosone/s/figures>

You may also use PLOS’s free figure tool, NAAS, to help you prepare publication quality figures: <https://journals.plos.org/plosone/s/figures#loc-tools-for-figure-preparation>.  NAAS will assess whether your figures meet our technical requirements by comparing each figure against our figure specifications.
