# Supplementary material for: Association between socioeconomic status and cardiovascular disease by sex: Mediating roles of psychological and behavioral factors
Source: PLoS One. 2026 Apr 1;21(4):e0345573. doi: 10.1371/journal.pone.0345573 (PMC13042698; doi:10.1371/journal.pone.0345573)
Supplement: Attachment — Submitted filename: Response_to_reviewers_auresp_2.docx [file pone.0345573.s019.docx]

PONE-D-25-52168R1

Association between socioeconomic status and cardiovascular disease by sex: mediating roles of psychological and behavioral factors

PLOS One

Dear Dr. Cho,

Thank you for submitting your manuscript to PLOS ONE. After careful consideration, we feel that it has merit but does not fully meet PLOS ONE’s publication criteria as it currently stands. Therefore, we invite you to submit a revised version of the manuscript that addresses the points raised during the review process.

Please submit your revised manuscript by Feb 20 2026 11:59PM. If you will need more time than this to complete your revisions, please reply to this message or contact the journal office at [plosone@plos.org](mailto:plosone@plos.org). When you're ready to submit your revision, log on to https://www.editorialmanager.com/pone/ and select the 'Submissions Needing Revision' folder to locate your manuscript file.

Please include the following items when submitting your revised manuscript:

- A letter that responds to each point raised by the academic editor and reviewer(s). You should upload this letter as a separate file labeled 'Response to Reviewers'.
- A marked-up copy of your manuscript that highlights changes made to the original version. You should upload this as a separate file labeled 'Revised Manuscript with Track Changes'.
- An unmarked version of your revised paper without tracked changes. You should upload this as a separate file labeled 'Manuscript'.

If you would like to make changes to your financial disclosure, please include your updated statement in your cover letter. Guidelines for resubmitting your figure files are available below the reviewer comments at the end of this letter.

If applicable, we recommend that you deposit your laboratory protocols in protocols.io to enhance the reproducibility of your results. Protocols.io assigns your protocol its own identifier (DOI) so that it can be cited independently in the future. For instructions see: <https://journals.plos.org/plosone/s/submission-guidelines#loc-laboratory-protocols>. Additionally, PLOS ONE offers an option for publishing peer-reviewed Lab Protocol articles, which describe protocols hosted on protocols.io. Read more information on sharing protocols at <https://plos.org/protocols?utm_medium=editorial-email&utm_source=authorletters&utm_campaign=protocols>.

We look forward to receiving your revised manuscript.

Kind regards,

Belal Hossain, PhD
Academic Editor
PLOS One

**Journal Requirements:**

**1. If the reviewer comments include a recommendation to cite specific previously published works, please review and evaluate these publications to determine whether they are relevant and should be cited. There is no requirement to cite these works unless the editor has indicated otherwise.**

In accordance with the editor’s comments, we reviewed and added literature showing that the strength of the association between socioeconomic status (SES) and cardiovascular disease (CVD) differs by sex, and that mediating factors such as smoking and psychological influences may operate differently in men and women. We also examined the literature on recommendations for presenting analyses of effect modification/interaction, and incorporated values for effect modification on both the additive and multiplicative scales into the manuscript.

**Additional Editor Comments:
The manuscript is well written, and the reviewers were satisfied with the revised version. However, there are some major points that need to be addressed before I consider this paper for acceptance.**

We deeply appreciate to carefully reading our text. We have now carefully considered the editor’s comments and reflected them in the revised manuscript to improve precision.

**1. The study conclusion should be revised (in both the Abstract and the Discussion section). The conclusion should be based on the findings of the current work rather than on hypothetical assumptions/recommendations.**

Thank you for your valuable comment. We have revised the Abstract and Discussion conclusions to focus strictly on our findings. The revised conclusion now avoids hypothetical recommendations and emphasizes the interpretive significance of our results.

[Page 2 Lines 28-31] Our findings indicate that psychological factors partially mediate the association between low SES and CVD among women, highlighting sex‑specific pathways in socioeconomic health disparities and underscoring the importance of incorporating mental health considerations into cardiovascular prevention strategies.

[Page 21 Lines 372-378] This study examined the relationship between SES and CVD incidence and explored the indirect effects of psychological factors and health behavior. We observed a sex-specific difference in the association between SES and CVD, with depressed mood and perceived anxiety/depression serving as a partial mediator among women. This suggests that differences in the distribution of psychological factors across SES subgroups contribute to CVD risk, providing insight into the mechanisms underlying socioeconomic inequalities in CVD. Our findings underscore the broader importance of considering psychological pathways in cardiovascular prevention strategies among low‑SES women.

**2. The rationale for sex-specific estimates is thin. The authors did not justify presenting sex-based estimates.**

In the revised manuscript, we have summarized previous studies to highlight that analyses conducted separately by sex demonstrate differences in the influence of mediating factors between men and women. In addition, we have incorporated a new paragraph reviewing the literature showing that the strength of the SES–CVD association varies by sex, and that mediating factors such as smoking and psychological influences may operate differently across men and women.

[Page 4 Lines 65-72] Previous studies have explored psychological and behavioral mediators in the SES–CVD association. Depression was reported to account for 10.9% of this relationship, although the cross-sectional design limited causal inference [19]. Cigarette smoking and physical inactivity explained part of the association between SDOH and CVD mortality, but reliance on a composite SDOH score obscured the specific contributions of individual attributes [20]. Other research has identified smoking, physical inactivity, alcohol use, and body mass index (BMI) as significant mediators of the association between SES and ischemic heart disease mortality in both men and women; however, the proportions mediated differed by sex [21].

[Page 4 Lines 73-84] The association between SES and CVD, as well as the mediating factors, appears to differ between men and women. Low SES has been linked to higher CVD risk, with this trend more pronounced in women [22, 23]. This is because women with lower SES tend to exhibit a worse profile of cardiovascular biomarkers [24]; however, the influence of SES on factors such as diet, physical activity, and psychosocial aspects may also differ by sex [22]. Importantly, the pathways through which SES influences CVD may differ in strength between men and women. For example, smoking substantially contributes to socioeconomic inequalities in ischemic heart disease mortality, accounting for a larger proportion among men (29%) than women (16%) [21]. In contrast, psychosocial factors—particularly depression and stress—along with behavioral and lifestyle influences, disproportionately affect women, with depression being approximately twice as prevalent in women as in men, thereby increasing their risk of CVD [25]. Therefore, these observations underscore the need for sex-specific mediation analyses to better understand the underlying pathways.

**3. It is unclear whether all analyses were conducted separately for males and females.**

As the editor pointed out, the sex-specific analyses in this study are not clearly presented. In the LCA, SES was derived from the overall population and then stratified by sex, after which mediation analyses were conducted separately for men and women. In this study, the sample consisted of 5,034 men and 6,231 women. When LCA was applied to the overall population (original version), men were classified as 12.6% low SES, 63.2% medium SES, and 24.2% high SES, whereas women were classified as 17.5% low SES, 71.4% medium SES, and 11.0% high SES.

We subsequently examined whether the measurement of latent classes was invariant across sex (1). A chi-square difference test, based on the differences in two G² statistics and their associated degrees of freedom, indicated a p-value less than 0.05, confirming that class membership probabilities differed significantly between men and women. Therefore, because measurement invariance did not hold across gender, we conducted LCA separately for men and women.

(1) <https://support.sas.com/resources/papers/proceedings16/5500-2016.pdf>

We revised the overall analysis by conducting LCA separately for men and women, and specified this procedure in the Methods section. When LCA was conducted separately for men and women, high SES in women was characterized by non-working status, which differed from men. However, the analysis showed similar results, as among women, low SES was associated with a shorter average survival time until CVD, partially mediated by depressed mood and by perceived anxiety/depression.

[Page 2 Lines 20-21] SES was derived using latent class analysis based on four variables: income, education, working status, and health insurance, conducted separately by sex.

[Pages 6-7 Lines 131-133] The exposure variable was SES classified as high, medium, and low, based on LCA using four variables—self-reported household income, education level, working status, and health insurance—conducted separately for men and women.

[Page 8 Lines 176-178] We determined the number of latent classes (ranging from two to five) based on model fit statistics and theoretical interpretability in analyses conducted separately for men and women (S1 Table).

[Pages 8-9 Lines 182-184] The three-class model for both men and women demonstrated the best fit based on AIC, BIC, and G² values. Its entropy values (0.63 for men and 0.69 for women) further indicate good class interpretability and separation.

[Page 9 Lines 190-194] The SES-specific curves for both sexes were non-parallel with differing slopes, indicating a violation of the proportional hazards assumption (S1-S2 Figs). However, their approximately linear form supported the Weibull assumption, justifying the use of an AFT model. The AFT model was formulated using survival functions, with the Weibull distribution showing the best fit based on AIC criteria in both men and women (S2 Table).

[Page 12 Lines 249-258] We evaluated the characteristics of each latent class separately for men and women based on mean posterior and item-response probabilities (Table 2). In men, latent class 1 (n = 639, 12.7%) was classified as low SES, reflecting low income, low education, and non-working status. Class 2 (n = 3,177, 63.1%) represented medium SES, with intermediate levels of income and education. Class 3 (n = 1,218, 24.2%) corresponded to high SES, characterized by high income, higher education, and full-time employment. In women, latent class 1 (n = 4,471, 71.8%) represented medium SES, with intermediate income and education. Class 2 (n = 685, 11.0%) corresponded to high SES, defined by high income and higher education. Class 3 (n = 1,075, 17.3%) was classified as low SES, reflecting low income, low education, and non-working status. In all analyses, the reference category was set as medium SES, as it had the largest sample size and allowed for the assessment of trends across low and high SES groups.

[Page 12 Lines 259-260] Table 2. Mean posterior and item-response probabilities from sex-specific three-class latent class models.

[Page 13 Line 262] Table 3 presents the distribution of SES obtained from sex-specific LCA.

[Page 19 Lines 307-313] This study used LCA based on household income, education, work status, and health insurance to classify SES into low, medium, and high, providing a more detailed profile than traditional indicators. Among men, those in the high SES group were typically classified as having full-time working status, whereas among women, the high SES group was characterized by having no working status. This suggests sex-specific differences in the composition of SES: among women, even those in higher SES groups may rely less on personal earnings from labor, with income instead derived from household or external resources.

**4. It is unclear how the 'proportion mediated' was calculated. The author cited a reference, but it was for odds ratios, whereas the present study used AFT models.**

As the reviewer correctly pointed out, the cited reference pertained to odds ratios and was therefore inappropriate for our study. We verified the correct procedure using the macro we employed and have now revised the manuscript to include a detailed description of how the proportion mediated was calculated.

[Pages 9-10 Lines 208-211] The proportion mediated (PM) was calculated on the mean survival ratio scale, as implemented in the SAS macro for causal mediation analysis [39]. Given that the TE in an AFT model decomposes multiplicatively into the NDE and NIE (i.e., TE = NDE * times NIE), the PM was defined as [NDE * times (NIE - 1)] / (TE - 1).

**5. The authors mentioned 'interactions' by sex in many places. I assumed the authors were referring to 'effect modification'. In either case, the recommendations for presenting analyses of effect modification/interaction were not followed.**

We appreciate the reviewer’s insightful comment regarding the presentation of our interaction analysis. We used the term "interaction" because our analysis was based on VanderWeele’s mediation macro, which incorporates an exposure–mediator interaction term in the regression models (1, 2). In our study, we likewise included SES × mediator interaction terms in the Weibull regression models and evaluated the statistical significance of these interaction terms with p-values.

However, we agree that following the recommended guidelines for reporting effect modification and interaction enhances the clarity of our findings. Therefore, following the published recommendations by Knol and VanderWeele (2012), we evaluated the effect modification between SES and mediator variables on both additive and multiplicative scales (3). In this response letter, we provide the detailed results of these analyses, using depressed mood in the male population as a representative example. For men, the RERI (Additive Scale) for low and high SES were -0.31 (95% CI: -0.84, 0.22) and -0.42 (95% CI: -0.83, -0.01), respectively. The multiplicative TRs were 1.22 (95% CI: 0.75, 2.00) and 1.36 (95% CI: 0.53, 3.46). Accordingly, our evaluation of effect modification indicated that high SES was associated with agonistic effects on the additive scale, while no significant effects were observed on the multiplicative scale.

(1) Valeri, L., & VanderWeele, T. J. (2015). SAS macro for causal mediation analysis with survival data. *Epidemiology*, *26*(2), e23-e24.

(2) *Valeri, L., & VanderWeele, T. J. (2013). Mediation analysis allowing for exposure–mediator interactions and causal interpretation: theoretical assumptions and implementation with SAS and SPSS macros. Psychological methods, 18(2), 137.*

(3) Knol, M. J., & VanderWeele, T. J. (2012). Recommendations for presenting analyses of effect modification and interaction. *International journal of epidemiology*, *41*(2), 514-520.

|  | | Socioeconomic status (Men) | | | Time ratio (95%CI) for low SES within strata of depressed mood | Time ratio (95%CI) for high SES within strata of depressed mood |
| --- | --- | --- | --- | --- | --- | --- |
|  |  | low | medium | high |  |  |
|  |  | Time ratio (95%CI) | Time ratio (95%CI) | Time ratio (95%CI) |  |  |
| Depressed mood | Yes | 1.14 (0.76, 1.71) | 0.92 (0.71, 1.20) | 1.29 (0.53, 3.13) | 1.22 (0.76, 1.96) | 1.39 (0.55, 3.48) |
|  | No | 1.00 (0.86, 1.17) | 1.0 | 1.02 (0.84, 1.25) | 1.00 (0.86, 1.17) | 1.02 (0.84, 1.25) |
| Time ratio (95%CI) for depressed mood within strata of SES | | 1.13 (0.75, 1.72) | 0.93 (0.71, 1.20) | 1.26 (0.51, 3.09) |  |  |
| Measure of interaction on additive scale (95%CI) | | -0.31 (-0.84, 0.22) |  | -0.42 (-0.83, -0.01) |  |  |
| Measure of interaction on multiplicative scale (95%CI) | | 1.22 (0.75, 2.00) |  | 1.36 (0.53, 3.46) |  |  |

Notes: TRs are adjusted for age, residence, marital status, obesity, previous diabetes mellitus, and previous hypertension. The measure of effect modification on the additive scale was assessed using the Relative Excess Risk due to Interaction (RERI), calculated by converting TR into Hazard Ratios (HR) based on the parameter scale, and interpreting HR as an approximation of Risk Ratios (RR). Confidence intervals were estimated using the delta method.

We described effect modification more explicitly in the Methods and Results sections and added analyses of effects on both the multiplicative and additive scales to S3 Table.

[Page 10 Lines 213-219] For sensitivity analysis, we tested the effect modification on both the multiplicative and additive scales between SES and the binary mediators (S3 Table), in order to incorporate these findings into the causal mediation analysis. Effect modification was evaluated on the multiplicative scale using TRs derived from exponentiated regression coefficients. On the additive scale, it was assessed using the relative excess risk due to interaction (RERI), calculated by converting TRs into hazard ratios via the parameter scale and interpreting hazard ratios as an approximation of risk ratios. Confidence intervals were estimated using the delta method.

[Page 15 Lines 271-275] Multiplicative effects of mediators between SES and CVD showed no statistically significant associations in either men or women across all SES levels (S3 Table). On the additive scale, antagonistic effects were observed for high SES combined with depressed mood (in both men and women), perceived anxiety/depression in men, smoking status in women, and physical activity in women.

**S3 Table. Effect modification on multiplicative and additive scales between socioeconomic status and binary mediators by sex.**

| **Measure of effect modification on multiplicative scale** | | | | |
| --- | --- | --- | --- | --- |
|  | Men | | Women | |
|  | Time ratio  (95% CI) | P-value | Time ratio  (95% CI) | P-value |
| Low SES: having depressed mood | 1.22  (0.75, 2.00) | 0.422 | 1.16  (0.82, 1.65) | 0.390 |
| High SES: having depressed mood | 1.36  (0.53, 3.46) | 0.520 | Inf  (0, Inf) | 0.999 |
| Low SES: having perceived anxiety/depression | 1.01  (0.69, 1.49) | 0.951 | 1.00  (0.74, 1.35) | 0.998 |
| High SES: having perceived anxiety/depression | 1.35  (0.68, 2.67) | 0.389 | 0.89  (0.29, 2.72) | 0.845 |
| Low SES: ever smoker | 0.61  (0.36, 1.03) | 0.066 | 0.86  (0.54, 1.37) | 0.523 |
| High SES: ever smoker | 1.19  (0.79, 1.81) | 0.404 | Inf  (0, Inf) | 0.999 |
| Low SES: physical activity at least one day per week | 1.14  (0.84, 1.55) | 0.410 | 1.15  (0.85, 1.56) | 0.349 |
| High SES: physical activity at least one day per week | 0.98  (0.68, 1.41) | 0.911 | 2.25  (0.74, 6.83) | 0.152 |
| **Measure of effect modification on additive scale** | | | | |
|  | Men | | Women | |
|  | RERI | P-value | RERI | P-value |
| Low SES: having depressed mood | -0.31  (-0.84, 0.22) | 0.256 | -0.22  (-1.26, 0.82) | 0.680 |
| High SES: having depressed mood | -0.42  (-0.83, -0.01) | 0.044* | -1.30  (-2.14, -0.46) | 0.002** |
| Low SES: having perceived anxiety/depression | -0.04  (-0.78, 0.71) | 0.923 | 0.13  (-1.15, 1.40) | 0.844 |
| High SES: having perceived anxiety/depression | -0.50  (-0.88, -0.11) | 0.011* | 0.09  (-0.40, 0.58) | 0.720 |
| Low SES: ever smoker | 0.57  (-0.28, 1.42) | 0.190 | 0.50  (-0.01, 2.01) | 0.519 |
| High SES: ever smoker | -0.29  (-0.59, 0.00) | 0.052 | -1.15  (-2.12, -0.17) | 0.021* |
| Low SES: physical activity at least one day per week | -0.16  (-0.57, 0.25) | 0.446 | -0.25  (-0.99, 0.49) | 0.501 |
| High SES: physical activity at least one day per week | 0.04  (-0.19, 0.26) | 0.748 | -0.69  (-1.23, -0.16) | 0.011* |

***** p < .05. ** p < .01. ***p < .001.

Note: The model was adjusted for age, residence, marital status, obesity, previous diabetes mellitus, and previous hypertension. The measure of effect modification on the multiplicative scale is expressed as a Time Ratio (TR), derived by exponentiating the regression coefficients. The measure of effect modification on the additive scale was assessed using the Relative Excess Risk due to Interaction (RERI), calculated by converting TR into Hazard Ratios (HR) based on the parameter scale, and interpreting HR as an approximation of Risk Ratios (RR). Confidence intervals were estimated using the delta method.

**6. There should be at least one sensitivity analysis using a Cox proportional hazards model, as it is most frequently used for modeling survival outcomes.**

We agree with the editor’s comment that the Cox proportional hazards model is the most commonly used approach in survival analysis. However, since the proportional hazards assumption was not satisfied in our data for both men and women, we conducted the analysis using an AFT model instead. We acknowledge that this rationale was not clearly stated in the original manuscript, which may have led to confusion. To address this, we have added a detailed explanation in the Methods section and included the relevant results in S1-S2 Figs.

[Page 9 Lines 185-196] Survival analysis was performed to examine the association between SES and CVD, considering both the Cox proportional hazards model and the parametric accelerated failure time (AFT) model [38]. Log–log transformed survivor functions were evaluated to determine whether the event times across exposure groups followed a Weibull distribution and whether the hazard functions were proportional [39]. The SES-specific curves for both sexes were non-parallel with differing slopes, indicating a violation of the proportional hazards assumption (S1-S2 Figs). However, their approximately linear form supported the Weibull assumption, justifying the use of an AFT model. The AFT model was formulated using survival functions, with the Weibull distribution showing the best fit based on AIC criteria in both men and women (S2 Table). Time was defined as the duration from baseline enrollment to the first occurrence of CVD, death, or the end of follow‑up. The participants were censored by a CVD event, death, or 31 December 2018, whichever occurred first.

**S1 Fig. Log–log plot of estimated survivor functions by SES group in men.** Log of the negative log of the estimated survivor function plotted against the log of follow-up duration for men, stratified by SES group (three categories).

**S2 Fig. Log–log plot of estimated survivor functions by SES group in women.** Log of the negative log of the estimated survivor function plotted against the log of follow-up duration for women, stratified by SES group (three categories).

In addition, since the proportional hazards assumption was not satisfied, we did not upload the results as a separate supplementary file; however, we have included the mediation analysis results using the Cox proportional hazards model as follows:

| Mediator | SES | Natural direct effect | | Natural indirect effect | | Total effect | | PM |
| --- | --- | --- | --- | --- | --- | --- | --- | --- |
|  |  | Hazard ratio | 95% CI | Hazard ratio | 95% CI | Hazard ratio | 95% CI |  |
| **Men** |  |  |  |  |  |  |  |  |
| Depressed mood | | | | | | | | |
|  | Low | 0.92 | 0.73, 1.16 | 1.00 | 0.98, 1.03 | 0.92 | 0.73, 1.16 | -5.3% |
|  | Medium | 1.00 (ref) |  | 1.00 (ref) |  | 1.00 (ref) |  |  |
|  | High | 1.01 | 0.75, 1.36 | 1.00 | 0.99, 1.00 | 1.01 | 0.75, 1.36 | -14.5% |
| Perceived anxiety/depression | | | | | | | | |
|  | Low | 0.91 | 0.72, 1.15 | 1.02 | 1.00, 1.04 | 0.93 | 0.74, 1.16 | -19.4% |
|  | Medium | 1.00 (ref) |  | 1.00 (ref) |  | 1.00 (ref) |  |  |
|  | High | 1.01 | 0.75, 1.36 | 1.00 | 0.99, 1.00 | 1.01 | 0.75, 1.36 | -28.3% |
| Smoking status | | | | | | | | |
|  | Low | 0.92 | 0.73, 1.15 | 1.01 | 1.00, 1.02 | 0.92 | 0.73, 1.16 | -6.9% |
|  | Medium | 1.00 (ref) |  | 1.00 (ref) |  | 1.00 (ref) |  |  |
|  | High | 1.01 | 0.75, 1.36 | 1.00 | 0.98, 1.01 | 1.01 | 0.75, 1.36 | -32.2% |
| Physical activity | | | | | | | | |
|  | Low | 0.89 | 0.71, 1.12 | 1.03* | 1.00, 1.07 | 0.92 | 0.73, 1.16 | -38.4% |
|  | Medium | 1.00 (ref) |  | 1.00 (ref) |  | 1.00 (ref) |  |  |
|  | High | 1.02 | 0.76, 1.37 | 0.99 | 0.97, 1.00 | 1.01 | 0.75, 1.36 | -197.4% |
| **Women** |  |  |  |  |  |  |  |  |
| Depressed mood | | | | | | | | |
|  | Low | 1.28* | 1.06, 1.56 | 1.03* | 1.00, 1.05 | 1.32** | 1.09, 1.60 | 10.3% |
|  | Medium | 1.00 (ref) |  | 1.00 (ref) |  | 1.00 (ref) |  |  |
|  | High | 0.81 | 0.45, 1.47 | 0.99 | 0.98, 1.00 | 0.80 | 0.44, 1.46 | 3.4% |
| Perceived anxiety/depression | | | | | | | | |
|  | Low | 1.28* | 1.06, 1.55 | 1.03** | 1.01, 1.06 | 1.32** | 1.09, 1.61 | 13.0% |
|  | Medium | 1.00 (ref) |  | 1.00 (ref) |  | 1.00 (ref) |  |  |
|  | High | 0.81 | 0.45, 1.47 | 1.01 | 0.99, 1.02 | 0.81 | 0.45, 1.48 | -3.1% |
| Smoking status | | | | | | | | |
|  | Low | 1.31** | 1.08, 1.59 | 1.01 | 0.999, 1.03 | 1.33** | 1.09, 1.61 | 5.4% |
|  | Medium | 1.00 (ref) |  | 1.00 (ref) |  | 1.00 (ref) |  |  |
|  | High | 0.80 | 0.44, 1.46 | 1.00 | 0.98, 1.01 | 0.80 | 0.44, 1.45 | 1.8% |
| Physical activity | | | | | | | | |
|  | Low | 1.31** | 1.08, 1.58 | 1.01 | 0.996, 1.03 | 1.32** | 1.09, 1.60 | 5.1% |
|  | Medium | 1.00 (ref) |  | 1.00 (ref) |  | 1.00 (ref) |  |  |
|  | High | 0.80 | 0.44, 1.45 | 1.00 | 0.99, 1.00 | 0.80 | 0.44, 1.44 | 0.4% |

***** p < .05. ** p < .01. ***p < .001. Note: The model was adjusted by age, residence, marital status, obesity, previous diabetes mellitus, and previous hypertension.

**7. The authors cited a reference, but I strongly recommend describing which mediation analysis technique was used, such as the counterfactual framework-based weighting approach.**
We appreciate the reviewer’s suggestion to clarify the mediation analysis technique. In the revised manuscript, we have explicitly described the analytic framework: mediation was conducted within a counterfactual approach by combining a logistic regression model for the binary mediator with an accelerated failure time (AFT) model for the survival outcome, allowing for exposure–mediator interactions. These details have been added and the Methods section reorganized accordingly.

[Pages 9-10 Lines 197-211] Causal mediation analysis provides a more general and rigorous framework for survival models, accommodating nonlinear relationships and interactions [37]. Specifically, to evaluate the causal pathways via the binary mediator, we performed mediation analysis within a counterfactual framework by integrating a logistic regression model for the mediator’s conditional probability and an AFT model for the survival outcome, allowing for exposure–mediator interactions [38]. The total effect (TE) was decomposed into the natural direct effect (NDE), reflecting the exposure’s effect independent of the mediator, and the natural indirect effect (NIE), representing the effect transmitted through exposure induced changes in the binary mediator and their impact on survival under the AFT model. Causal effects were interpreted on the mean survival ratio scale, with time ratios (TRs) < 1 denoting reduced survival and TRs > 1 denoting increased survival, thereby quantifying acceleration or deceleration of event time. The proportion mediated (PM) was calculated on the mean survival ratio scale, as implemented in the SAS macro for causal mediation analysis [39]. Given that the TE in an AFT model decomposes multiplicatively into the NDE and NIE (i.e., TE = NDE $\times$ NIE), the PM was defined as [NDE $\times$ (NIE $-$ 1)] / (TE $-$ 1).

[Note: HTML markup is below. Please do not edit.]

**Reviewers' comments:**

Reviewer's Responses to Questions

**Comments to the Author**

1. If the authors have adequately addressed your comments raised in a previous round of review and you feel that this manuscript is now acceptable for publication, you may indicate that here to bypass the “Comments to the Author” section, enter your conflict of interest statement in the “Confidential to Editor” section, and submit your "Accept" recommendation.

Reviewer #1: All comments have been addressed

Reviewer #3: All comments have been addressed

2. Is the manuscript technically sound, and do the data support the conclusions?

The manuscript must describe a technically sound piece of scientific research with data that supports the conclusions. Experiments must have been conducted rigorously, with appropriate controls, replication, and sample sizes. The conclusions must be drawn appropriately based on the data presented.

Reviewer #1: Yes

Reviewer #3: Yes

3. Has the statistical analysis been performed appropriately and rigorously?

Reviewer #1: Yes

Reviewer #3: Yes

4. Have the authors made all data underlying the findings in their manuscript fully available?

The [PLOS Data policy](http://www.plosone.org/static/policies.action#sharing) requires authors to make all data underlying the findings described in their manuscript fully available without restriction, with rare exception (please refer to the Data Availability Statement in the manuscript PDF file). The data should be provided as part of the manuscript or its supporting information, or deposited to a public repository. For example, in addition to summary statistics, the data points behind means, medians and variance measures should be available. If there are restrictions on publicly sharing data—e.g. participant privacy or use of data from a third party—those must be specified.

Reviewer #1: Yes

Reviewer #3: No

5. Is the manuscript presented in an intelligible fashion and written in standard English?

PLOS ONE does not copyedit accepted manuscripts, so the language in submitted articles must be clear, correct, and unambiguous. Any typographical or grammatical errors should be corrected at revision, so please note any specific errors here.

Reviewer #1: Yes

Reviewer #3: Yes

6. Review Comments to the Author

Please use the space provided to explain your answers to the questions above. You may also include additional comments for the author, including concerns about dual publication, research ethics, or publication ethics. (Please upload your review as an attachment if it exceeds 20,000 characters)

Reviewer #1: The authors have satisfactorily addressed all comments from the previous review. The revised manuscript is clearer and methodologically sound, with improved description of study design, mediator definitions, and causal mediation assumptions.

The statistical analyses are appropriate and sufficiently detailed, and the added sensitivity analyses support the robustness of the findings. The discussion has been strengthened, particularly in interpreting sex-specific results and policy implications, and the limitations are adequately acknowledged.

Overall, the manuscript meets the technical and scientific standards of PLOS ONE and is acceptable for publication in its current form.

Reviewer #3: I would like to inform you that all reviewer comments have been fully addressed. The manuscript has been revised accordingly and is now ready for publication.

7. PLOS authors have the option to publish the peer review history of their article ([what does this mean?](https://journals.plos.org/plosone/s/editorial-and-peer-review-process#loc-peer-review-history)). If published, this will include your full peer review and any attached files.
If you choose “no”, your identity will remain anonymous but your review may still be made public.
**Do you want your identity to be public for this peer review?** For information about this choice, including consent withdrawal, please see our [Privacy Policy](https://www.plos.org/privacy-policy).

Reviewer #1: **Yes:** Faisal Alkulaib

Reviewer #3: No

[NOTE: If reviewer comments were submitted as an attachment file, they will be attached to this email and accessible via the submission site. Please log into your account, locate the manuscript record, and check for the action link "View Attachments". If this link does not appear, there are no attachment files.]

To ensure your figures meet our technical requirements, please review our figure guidelines: https://journals.plos.org/plosone/s/figures

You may also use PLOS’s free figure tool, NAAS, to help you prepare publication quality figures: https://journals.plos.org/plosone/s/figures#loc-tools-for-figure-preparation.

NAAS will assess whether your figures meet our technical requirements by comparing each figure against our figure specifications.
